# Spatio-temporal distribution of soil microbial communities and nutrient availability around a municipal solid waste landfill

**DOI:** 10.3389/fmicb.2025.1583149

**Published:** 2025-06-16

**Authors:** Mingye Zhan, Yanqiu Sun, Huanjie Lan, Tao Zhou, Youcai Zhao, Li Yang

**Affiliations:** ^1^Chengdu Huantou Urban Management Services Co., Ltd, Chengdu, China; ^2^College of Environmental Science and Engineering, Tongji University, Shanghai, China

**Keywords:** landfill surrounding environment, microbial diversity, spatial distribution, soil nutritional index, functional gene

## Abstract

**Introduction:**

The landfills may have notable ecological impacts on the surrounding environment, yet little is known about the microbial community and nutrient conditions in surrounding soil.

**Methods:**

Using high-throughput sequencing technology, we analyzed the spatio-temporal distribution of bacterial and fungal communities in soils surrounding a large-scale landfill. The component of landfill waste and twelve soil properties were detected, including four nutritional indices and eight heavy metal elements.

**Results and discussion:**

Our results revealed landfill-specific enrichment of bacterial genera *Pseudomonas* (0.13–6.43%), *Marmoricola* (0.12–4.82%), *Sphingomonas* (0.64–5.24%), and *Nocardioides* (0.51–6.3%) and fungal genera *Alternaria* (0.23–12.85%), *Pyrenochaetopsis* (0.028–10.12%) and Fusarium (0.24–4.07%). Their relative abundances exhibited significant variations across landfill age gradients and soil depth profiles (*p* ≤ 0.05). Random forest and structural equal models (SEM) confirmed the direct correlation between soil TOC, heavy metals including Cu, Cd and Pb and microbial diversity. While soil heavy metals mainly exhibited negative effects on microbial diversity, dominant microbial taxa such as *Lysobacter, Nocardioides, Pseudopithomyces*, and *Chaetomium* showed potential tolerance to heavy metal stress in soil around the landfill. In soil around the landfill, higher concentrations of total nitrogen (TN), available phosphorus (AP) and available potassium (AK) were observed in the upper layers near the aged landfill areas, whereas higher concentration of total organic carbon (TOC) were detected around fresh landfill area. The distribution of microbial taxa and predicted functional profiles were strongly associated with the nutrients availability. The findings revealed that landfill activities influenced the structure and function of microbial community, contributing to the complex spatio-temporal distribution of nutrients in the surrounding soil.

## 1 Introduction

Landfilling persists as the predominant global strategy for managing municipal solid waste (MSW), currently accounting for ~70% of urban waste disposal through landfills or open dumps. Projections indicate that urban solid waste generation will escalate to 3.5 billion tons by 2050 (Vyas et al., 2022). At present, the United States (258 million tons), China (220 million tons), and India (169 million tons) are the top three MSW-generating nations (Nanda and Berruti, 2021). Despite technological advancements, the United Stated continues to landfill over 136 million tons of MSW annually, while 547 operational urban landfill facilities in China process ~161 million tons yearly. The heterogeneous nature of MSW renders landfills highly diverse terrestrial ecosystems in terms of microbial community structure (Zainun and Simarani, 2018). Previous studies have extensively characterized the microbial populations within landfill matrices, involving bacterial and fungal communities (Stamps et al., 2016). Core microbial communities play crucial roles in the decomposition of organic waste, with bacteria preferentially metabolizing degradable organic matter, while fungi specialize in recalcitrant organic matter degradation (Ma et al., 2022; Cai et al., 2024). It has been documented that the microbial community structure in deep and capped landfill sites differs from that in shallow, active landfill areas, containing a large number of methanogens and anaerobic bacteria involved in acidogenic fermentation, demonstrating spatial–temporal variations in microbial composition (Wang et al., 2017). Currently, there is a lack of research on the microbial community structure in soils surrounding landfills, and it remains unclear whether those microorganisms accumulate in adjacent soils and subsequently influence soil ecological functions.

Most studies have established a positive correlation between microbial diversity and ecosystem multifunctionality (Neher, 1999; Han et al., 2021; Chen et al., 2022; Sun et al., 2023). Microbial activity and interactions influence the cycling of carbon, nitrogen, and phosphorus in the soil and play a critical role in the bioremediation of heavy metal pollution in the soil, both of which are crucial for the stability of soil ecosystem (Jiao et al., 2019; Wang et al., 2023). Microorganisms decompose organic matter in the soil, providing energy sources for most soil organisms (Singh et al., 2020). The richness of soil biomass and interactions of microbial communities are key indicators of soil health (Sindhu et al., 2016). Soil organic carbon (OC) can be mineralized during microbial processes or sequestered as stable biochar. The accumulation of soil organic carbon enhances the aggregation of nutrients and microorganisms in the soil (Ma et al., 2022). Soil nutrient nitrogen (N) accumulates through microbial ammonification or nitrogen fixation (Khan et al., 2020), while it is also lost through physical and chemical pathways or microbial nitrification and denitrification (Hirsch and Mauchline, 2015). Bacteria and fungi in the soil secrete phosphatases and phytases or produce organic acids to dissolve organically bound phosphates, thereby increasing the release of nutrient phosphorus (P; Yadav et al., 2015). The structure and function of soil microbial communities play a decisive role in the soil nutrient level. There is a paucity of research on the impact of microbial community structures in soils surrounding landfills on soil fertility and nutrient cycling. Analyzing the organization and driving mechanisms of soil microbial communities surrounding landfills and their functional impacts on soil ecosystems is crucial for ecological restoration and environmental improvement of landfill sites.

Utilizing natural microbial diversity to maintain soil nutrients is an effective strategy for the ecological restoration of landfill sites and the improvement of soil health. To investigate the characteristics of microbial community structures and the influence of functional microbes on soil nutrient contents in areas surrounding landfills, this study collected soil samples from various depths (surface 0–0.5 m, shallow 0.5–2 m, and deep 2–4 m) around waste landfills of different ages (2 a, 19 a, 29 a). High-throughput sequencing technology was employed to analyze the spatio-temporal distribution patterns of bacterial and fungi communities in soil from areas surrounding landfill. Soil total organic carbon (TOC), total nitrogen (TN), available phosphorus (AP), and available potassium (AK) were measured as indicators of soil fertility. The composition of soil heavy metal element (Cu, Zn, Ni, Cr (VI), Hg, As, Pb, and Cd) and the composition of landfill waste were also assessed. The influence of environmental factors on microbial community was further discussed, and the microbial functional prediction analysis was conducted to preliminarily explore the mechanisms by which microorganisms affect soil nutrient content.

## 2 Materials and methods

### 2.1 Brief situation of research area

The landfill in this study is located in a hilly terrain valley in Sichuan Chengdu, southwest of China. According to the construction progress, the landfill is divided into three areas named: Phase I, Phase II, and Phase III, each designated as a domestic waste landfill area. Phase I covers an area of 55.73 ha, with a design capacity of 11.35 million m^3^, and was first put into operation in 1993; Phase II covers an area of 48.20 ha, with a design capacity of 23.4 million m^3^, and was put into operation in 2003. Both phases I and II were temporarily closed in 2019 and have stopped landfill operation; they are currently only subject to routine maintenance. Phase III covers an area of 47.60 ha and was put into service after completion in June 2020. Sampling for this study was conducted in September 2022, until when the landfill age of Phase I was 29 years old, the landfill age of Phase II was nearly 19 years old, and the landfill age of Phase III was 2 years old.

### 2.2 Sample collection

**Figure 1:**
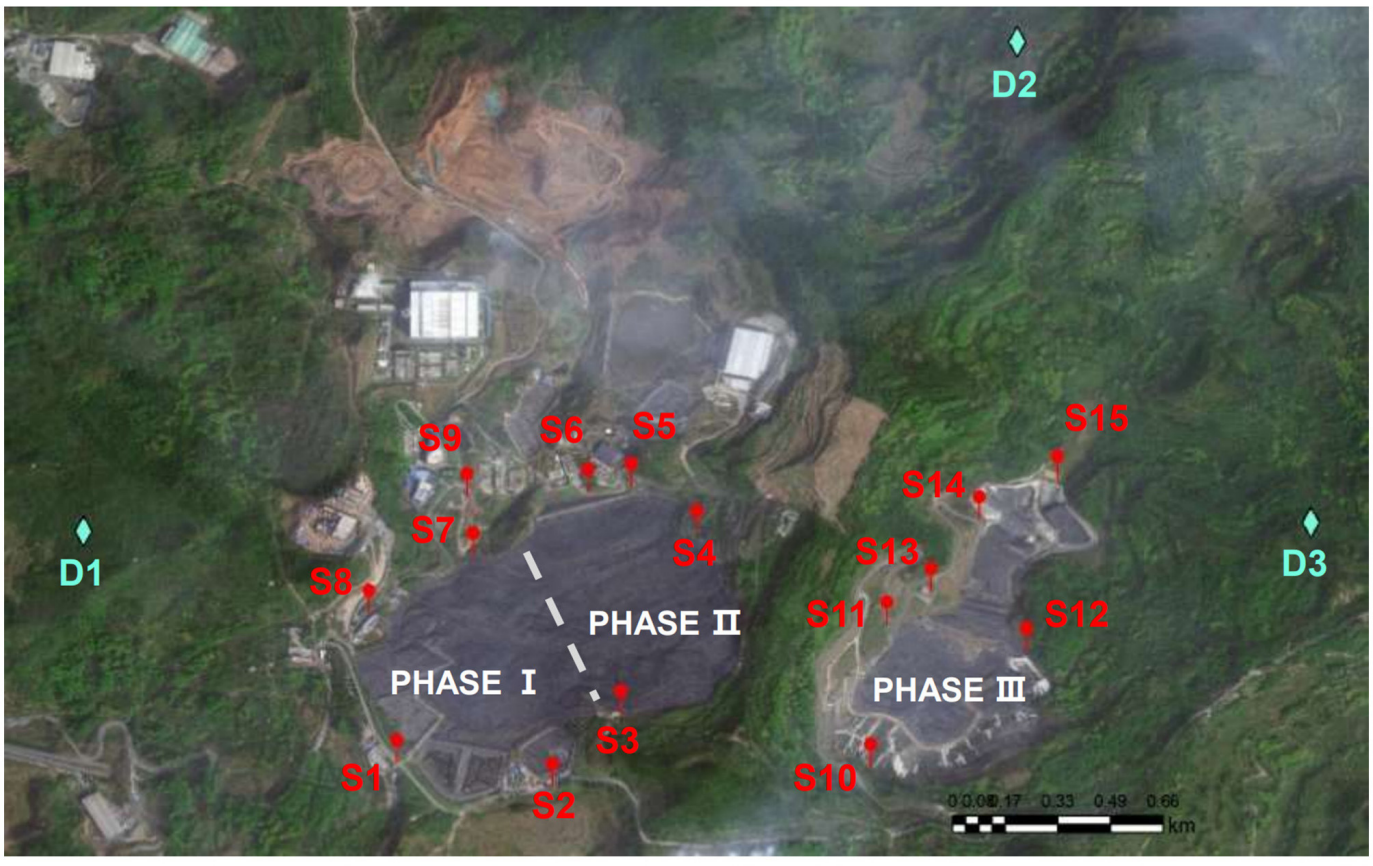
Information of soil and microorganism samples.

Soil samples were collected from areas surrounding the landfills of different ages (2 a, 19 a, 29 a), with sampling depths of 0–0.5 m and 0.5–2 m (shallow layer) and 2–4 m and 4–6 m (deep layer). A total of 15 sampling points (S1–S15) were set up around the landfill areas. At the same time, three control groups (D1–D3) were deployed in the external area upwind of the dominant wind direction and upstream of groundwater flow. A total of 5–20 subsamples were mixed in equal amounts to form 1 mixed sample, and a total of 59 samples were formed. ArcGIS Pro was used to align the geographic coordinates based on the 1:15,000 topographic map of the landfill site as a baseline (Figure 1). The detail of sample pretreatment is recorded in Supplementary material.

### 2.3 Landfill waste components and adjacent soil property measurement

Soil fertility indices, including total organic carbon (TOC), total nitrogen (TN), available phosphorus (AP), and available potassium (AK), were measured. The soil TOC was measured according to Soil-Determination of Organic Carbon-Potassium Dichromate Oxidation Spectrophotometric Method (HJ 615-2011) by visible light spectrophotometer (TL5, Shanghai YOKE Instrument Co., LTD, China). The soil TN was determined according to Soil quality-Determination of total nitrogen-Modified Kjeldahl method (HJ 717-2014). The soil AP was determined following the protocol of Soil quality-Determination of available phosphorus-Sodium hydrogen carbonate solution-Mo-Sb anti-spectrophotometric method (HJ704-2014) using an ultraviolet visible spectrophotometer (UV-1780, SHIMADZU, Japan). The soil AK was determined following the protocol of Potassium determination methods of forest soils (LY/T 1234-2015) using a flame photometer (FP6450, Shanghai Yidian Analytical Instrument Co., LTD., China). The soil samples were also analyzed for Cr (VI), Zn, Ni, and Cu contents using an atomic absorption spectrophotometer (TAS-990F, Beijing Puxi General Instrument Co., Ltd., China). The concentration of Hg and As was detected using an atomic fluorescence spectrophotometer (AFS-10B, Beijing Jitian Instrument Co., Ltd., China). A graphite spectrophotometer (TAS-990G, Beijing Puxi General Instrument Co., Ltd., China) was used to determine Pb and Cd. Landfill waste components were analyzed by manual classification and weighing.

### 2.4 DNA extraction and sequencing

Total microbial genomic DNA was extracted from 59 samples using the E.Z.N.A.^®^ soil DNA Kit (Omega Bio-tek, Norcross, GA, U.S.) following the manufacturer's instructions (Liu et al., 2016). The PCR product was extracted from 2% agarose gel and purified using the PCR Clean-Up Kit (YuHua, Shanghai, China) following the instructions of the manufacturer and quantified using Qubit 4.0 (Thermo Fisher Scientific, USA). Purified amplicons were pooled in equimolar amounts and paired-end sequenced on an Illumina MiSeq PE300 platform (Illumina, San Diego, USA) according to the standard protocols by Majorbio Bio-Pharm Technology Co. Ltd. (Shanghai, China).

### 2.5 Statistical analysis

Raw FASTQ files were de-multiplexed using an in-house Perl script, then quality-filtered with Fastp version 0.19.6 (https://github.com/OpenGene/fastp; Chen et al., 2018), and merged using Flash version 1.2.11 (Magoč and Salzberg, 2011). Bioinformatics analysis of the soil microbiota was carried out using the Majorbio Cloud platform (https://cloud.majorbio.com). The optimized sequences were clustered into operational taxonomic units (OTUs) using Uparse version 11 (http://www.drive5.com/uparse/; Stackebrandt and Goebel, 1994; Edgar, 2013), with 97% sequence similarity level. Based on the OTUs information, microbial diversity index calculation, taxonomic analysis, and functional genes annotation are recorded in Supplementary material. The data on microbial α-diversity in the soil samples were calculated with OriginPro 8 SR0 (version 8.0724). The R language vegan package was used to draw the OTU community bar charts. The Kruskal–Wallis rank-sum test was used to assess the differences between different sampling points. Environmental factor association analysis (db-RDA, Mantel test, and Spearman's correlation) was used to assess the correlations between dominant microbial genera and soil nutrients, heavy metal element contents, and the composition of landfill waste. The random forest and structural equation model (SEM) were established to analyze the potential driving mechanisms of the effects of soil characteristics on microbial diversity by “randomForest” and “piecewiseSEM” packages in R.

## 3 Results

### 3.1 Landfill waste components and adjacent soil properties

We collected waste samples at various depths (*n* = 72) from an aged landfill area that was closed 2 years ago. The landfill waste was primarily composed of rubber and plastic, textile, wood and bamboo, brick, tile and ceramic, humus soil, and others unclassified. The proportions of kitchen waste (~16.47%), paper (~2.28%), and wood and bamboo (~18.72%) were significantly higher at shallow depth (*p* ≤ 0.05), while the proportion of humus soil (~25.11%, *p* ≤ 0.05) significantly increased in the shallow-middle layers (0.5–2m). In addition, the rubber and plastic (34.57–46.86%) and textile (13.89–39.31%) waste maintained high proportions in deep layers (≥2 m). As the landfill depth increased, the landfill waste components have shown a vertical distribution pattern (Supplementary Figure S1).

**Figure 2:**
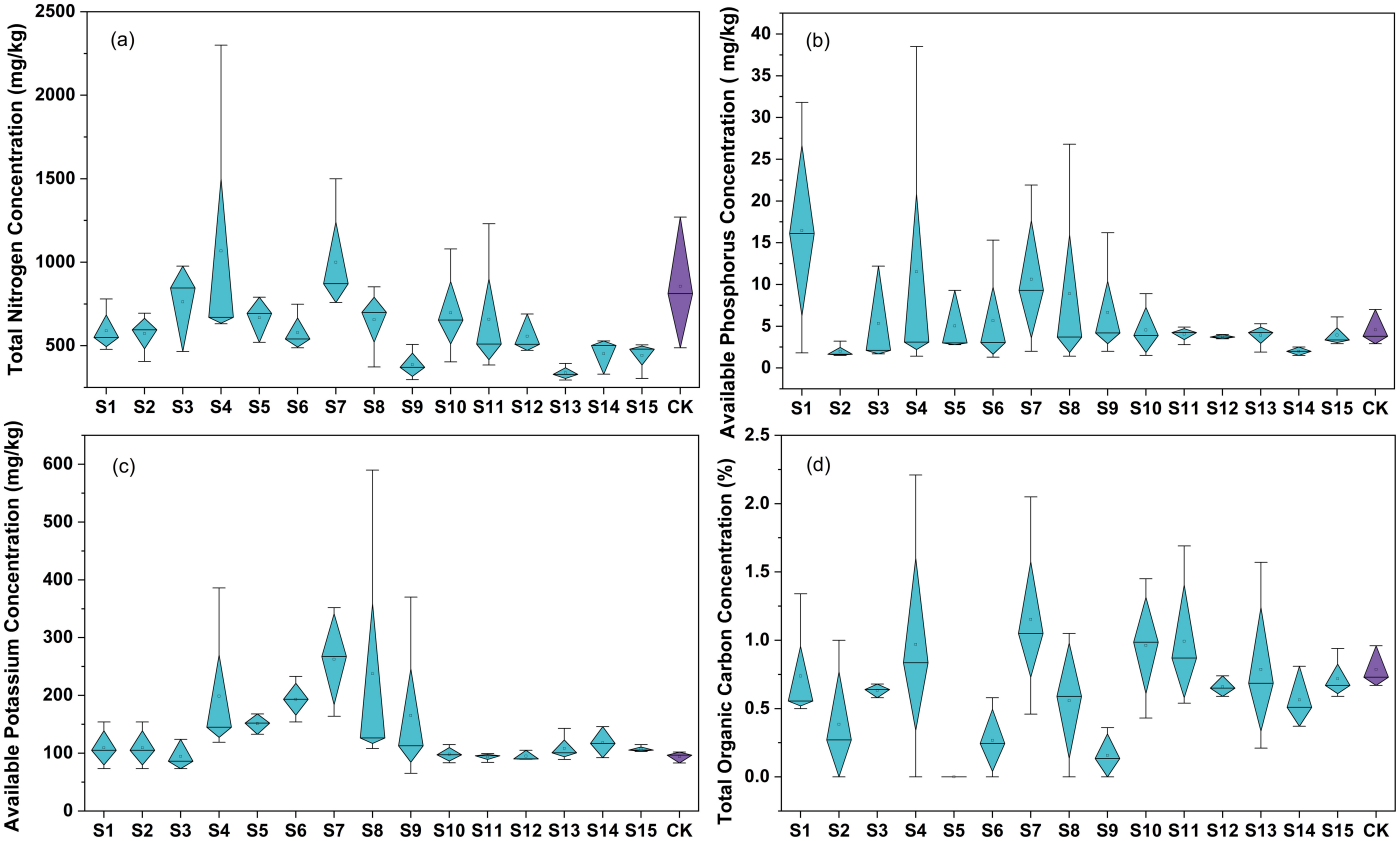
The content of soil nutritional indices in different samples. The X-axis represented samples in different sampling sites, the Y-axis represented the concentration of nutritional indices in soil. A one-way ANOVA was conducted to test the differences between groups, but no significant differences were found (*p* > 0.05). **(a)** TN (*p* = 0.07726), **(b)** AP (*p* = 0.50119), **(c)** AK (*p* = 0.10826), **(d)** TOC (*p* = 0.06489).

The adjacent soil nutrient analysis revealed distinct spatial patterns (Figure 2) compared with control points exhibiting mean values of 856.67 mg·kg^−1^ (TN), 4.57 mg·kg^−1^ (AP), 93.97 mg·kg^−1^ (AK), and 0.79% (TOC). The TOC and TN concentrations demonstrated significant depletion in soil samples surrounding the landfill (*p* ≤ 0.05), whereas AP and AK showed marked accumulation, primarily in the upper soil layers (≤2 m depth; Supplementary Figure S2). Soil nutrient contents varied with landfill age gradients. Soil TOC was higher near fresh landfill area, while TN, AP, and AK reached maximal concentrations in soils adjacent to the aged landfill area (19 a, 29 a; Figure 2).

This study also investigated the content of Cu, Zn, Ni, Cr (VI), Hg, As, Pb, and Cd in the adjacent soil. At most sampling sites surrounding the landfill, the concentration of Cu, Zn, Ni, and Cd did not exceed control points. In contrast, Cr (VI), Hg, As, and Pb showed higher concentration at sampling sites near aged landfill than the control value (without significance), indicating progressive accumulation of those heavy metal elements in the landfill surrounding environment (Supplementary Figure S3).

### 3.2 Microbial diversity profiling

**Figure 3:**
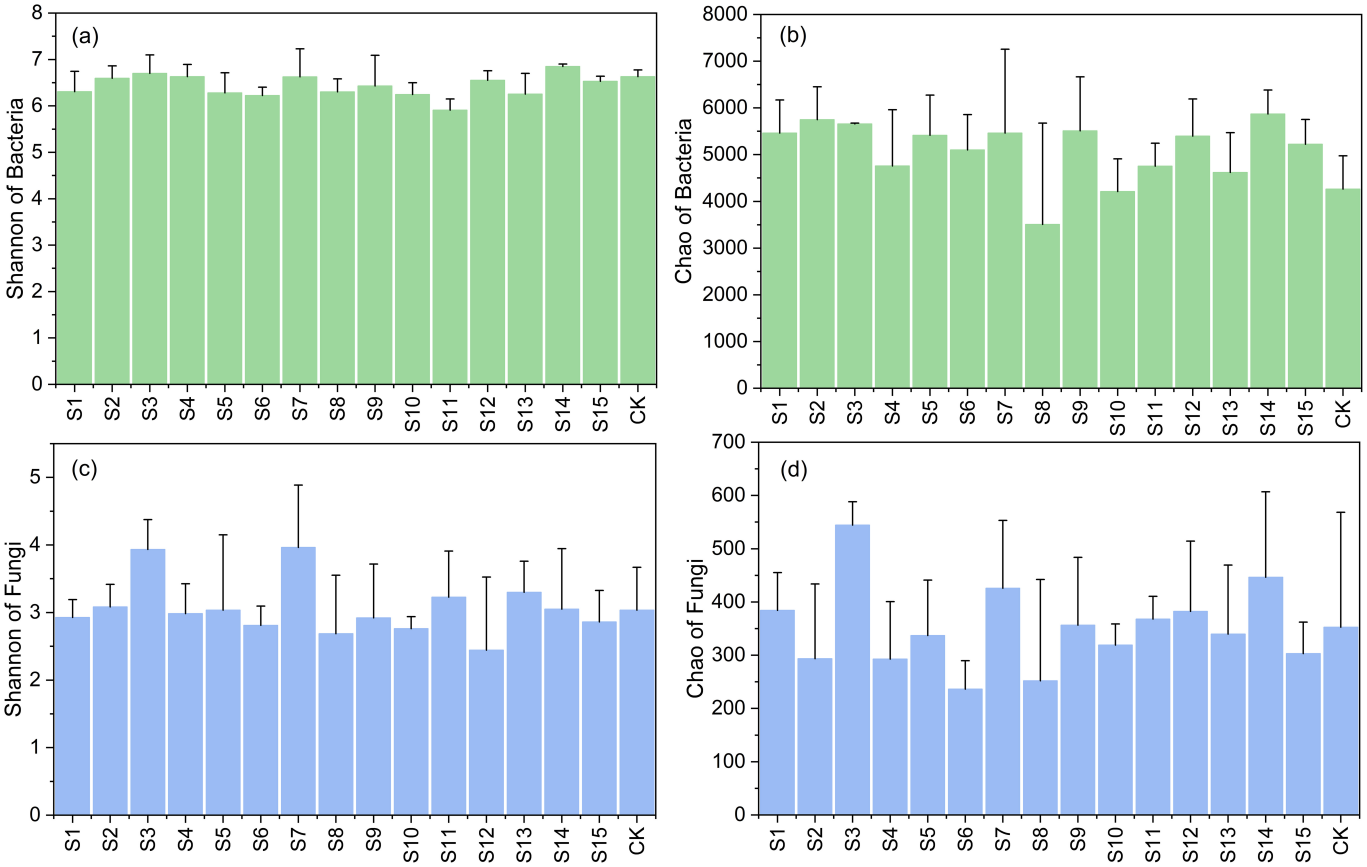
The bacterial and fungal Shannon index and Chao index in different samples. The X-axis represented samples in different sampling sites, the Y-axis represented the value of microbial α diversity indices. The green **(a, b)** presents bacterial diversity indices of each sample and the blue **(c, d)** presents fungal diversity indices of each sample. The index value reflects the average level of different depth at the sampling point. A one-way ANOVA was conducted to test the differences between groups, but no significant differences were found (*p* > 0.05).

In total, sequencing yielded 5,746,648 quality-filtered bacterial reads (55,807–122,313 per sample), clustered into 5,707 OTUs. As shown in Figure 3, landfill adjacent soils exhibited mean bacterial Shannon diversity of 6.41 vs. 6.63 at control sites, with 30% of the landfill samples exceeding control values. Bacterial richness (Chao index) reached an average of 5,079.42 in soil around landfill, surpassing average control values (4,264.00). The fungal analysis generated 4,180 OTUs from 4,228 to 161,484 reads per sample. Although the soil fungal Shannon indices surrounding landfill showed a marginal elevation (an average value of 3.06) compared to the average control values (3.03), ~45% of the landfill-adjacent soil samples had Chao1 values (average of 343.30) that exceeded the control values (average of 352.75).

### 3.3 Bacterial community architecture

**Figure 4:**
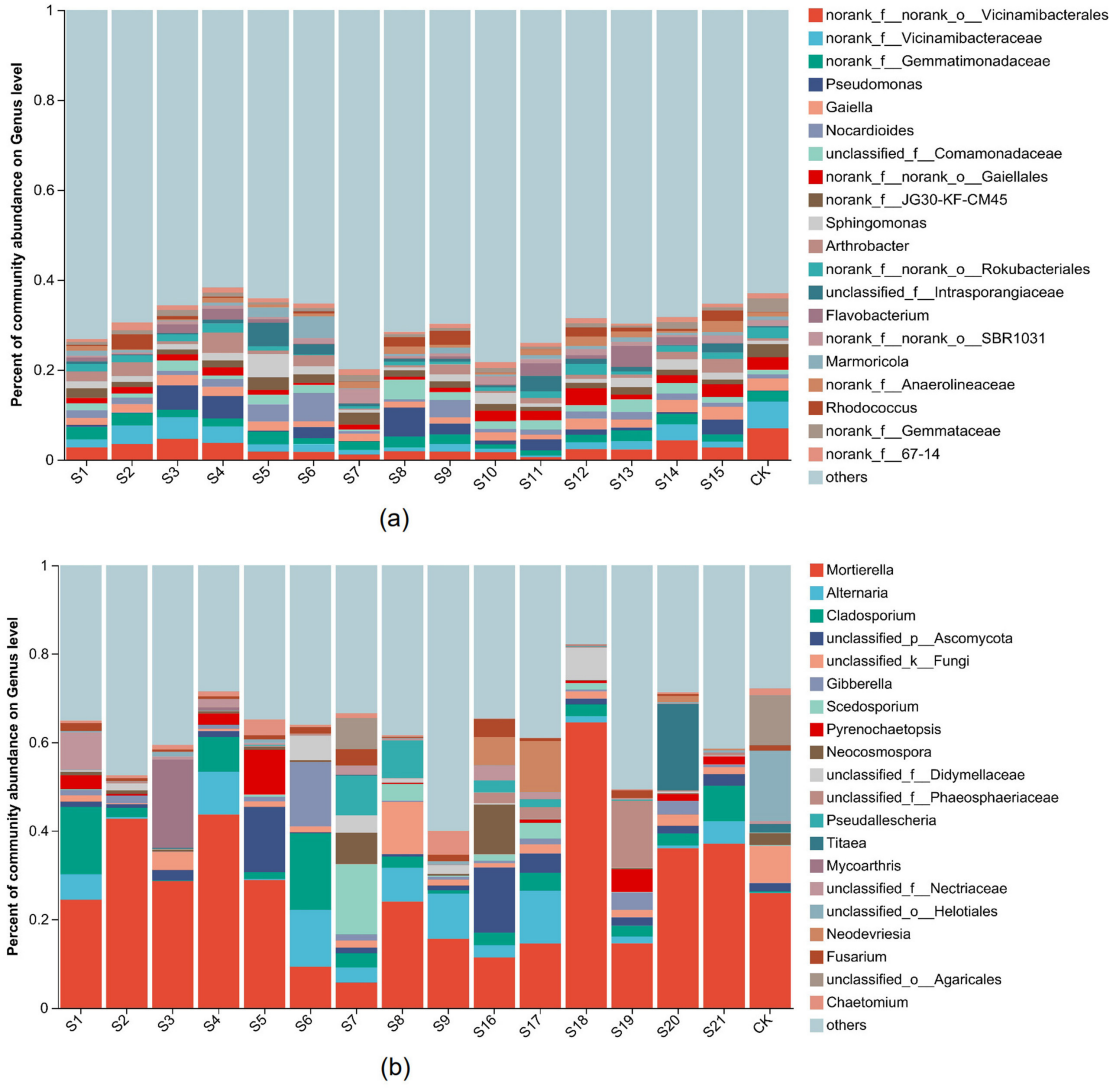
The top 20 dominant bacterial **(a)** and fungal **(b)** composition in different samples at genus level. The X-axis represented samples in different sampling sites, the Y-axis represented the relative abundance of dominant microbial populations. The relative abundance of each genus reflects the average level of different depth at the sampling point.

The composition of soil bacterial community surrounding the landfill is shown in Supplementary Figure S4a and Figure 4a. The results showed that, at the phylum level, the dominant bacterial phyla in soil control points were Proteobacteria (16.63%), Actinobacteriota (18.39%), Chloroflexi (14.35%), Acidobacteriota (20.22%), Planctomycetota (9.29%), and Bacteroidota (1.14%). In contrast, the dominant bacterial phyla in soil surrounding the landfill were Proteobacteria (20.19–40.08%), Actinobacteriota (15.67–34.42%), Chloroflexi (6.37–20.59%), Acidobacteriota (3.99–15.61%), Planctomycetota (2.55–7.41%), and Bacteroidota (1.43–8.75%; Supplementary Figure S4a). At the genus level, the dominant bacterial genera in soil control points were norank_f_Vicinamibacteraceae (6.94%) and Vicinamibacteraceae norank (6%), while the dominant bacterial genera in soil surrounding the landfill were norank_f_Vicinamibacteraceae (0.57–4.61%), *Pseudomonas* (0.13–6.43%), *Nocardioides* (0.51–6.3%), Comamonadaceae unclassified (0.34–4.37%), *Sphingomonas* (0.64–5.24%), and *Rhodococcus* (0.21–2.49%; Figure 4a).

The sampling points were classified according to the specific landfill age and divided into three groups (age2, age19, and age29). The dominant bacteria of soil in age2 samples were norank_f_Gemmatimonadaceae (2.62%), *Rhodococcus* (1.59%), and *Streptomyces* (1.13%). The dominant bacteria of soil in age19 samples were *Nocardioides* (3.53%), *Sphingomonas* (2.46%), *Pseudomonas* (2.12%), Intrasporangiaceae unclassified (2.21%), *Marmoricola* (2.21%), and *Lysobacter* (1.63%). The dominant bacteria of soil in age29 samples were norank_f_norank_o_Gaiellales (2.27%), norank_f_Anaerolineaceae (1.33%), *Flavobacterium* (1.79%), and *Ramlibacter* (1.46%; Supplementary Figure S5a).

The results of the assessment of differences among various sampling groups revealed significant variations in the relative abundance of specific microbial taxa across different age groups (*p* ≤ 0.05; Supplementary Figure S6a). In particular, the relative abundance of *Lacunisphaera* and *Leptospirillum* was significantly higher in age2 samples than that in age19 samples (*p* ≤ 0.05). The relative abundance of *Micromonospora*, Xanthomonadaceae unclassified, and *Pantoea* was significantly higher in age2 samples than that in age29 samples (*p* ≤ 0.05). The relative abundance of *Agromyces, Phyllobacterium*, and *Allorhizobium* was significantly higher in age19 samples than that in age29 samples (*p* ≤ 0.05). Conversely, the relative abundance of *Microterricola, Tolypothrix*, and *Emticicia* was significantly higher in age19 samples than that in age2 samples (*p* ≤ 0.05). Moreover, the relative abundance of *Levilinea, Ellin6067*, and Burkholderiales unclassified was significantly higher in age29 samples than which in age19 samples (*p* ≤ 0.05). Meanwhile, the relative abundance of *Anaerolinea, Methylosinus*, Sutterellaceae unclassified, and Bacteroidetes unclassified was significantly higher in age29 samples than that in age2 samples (*p* ≤ 0.05).

The sampling points were classified based on specific soil depths and divided into two categories, namely, the shallow layer (0–2 m) and the deep layer (2–6 m). In the shallow layer of soil around landfill, the dominant taxa included *Pseudomonas, Arthrobacter*, Intrasporangiaceae unclassified, and Anaerolineaceae norank, which exhibited higher relative abundances, while *Nocardioides, Gaiella*, Comamonadaceae unclassified, and *Marmoricola* had higher abundance in deep layer of soil surrounding landfill (Supplementary Figure S7a).

### 3.4 Fungal community architecture

The composition of soil fungal community around the landfill is illustrated in Supplementary Figure S4b and Figure 4b. At the phylum level, the dominant fungal phyla in soil control points were Ascomycota (44.2%), Mortierellomycota (25.91%), and Basidiomycota (15.78%), while the dominant fungal phyla in soil surrounding the landfill were Ascomycota (27.27–81.35%), Mortierellomycota (5.93–64.5%), and Basidiomycota (1.72–30%). At the genus level, the dominant fungal genera in soil control points were *Mortierella* (25.91%), Helotiales unclassified (15.92%), and Agaricales unclassified (11.37%), while the dominant fungal genera in soil surrounding the landfill were *Alternaria* (0.23–12.85%), *Cladosporium* (0.74–17.31%), and *Pyrenochaetopsis* (0.028–10.12%). In addition, a total of 1,926 unique fungal OTUs were identified in the soil surrounding the landfill, which were classified into 393 species (Supplementary Figure S8).

The sampling points were classified based on the specific landfill age and divided into three groups (age2, age19, and age29). The dominant fungi in the soil of age2 samples were *Scedosporium* (3.39%), *Pseudallescheria* (2.94%), and *Pleurotheciella* (3.44%). In the soil of age19 samples, the dominant fungi were *Alternaria* (7%), *Gibberella* (4.97%), *Pyrenochaetopsis* (3.12%), and *Mycoarthris* (3.27%). The dominant fungi in the soil of age29 samples were Phaeosphaeriaceae unclassified (3.85%), *Neodevriesia* (3.5%), and *Pseudopithomyces* (2.1%; Supplementary Figure S5b).

The results of tests assessing the differences among various sampling groups revealed significant differences in the relative abundance of specific fungal taxa across different age groups (*p* ≤ 0.05; Supplementary Figure S6b). The relative abundance of Saccharomycetales unclassified, *Verticillium*, and *Neodidymelliopsis* was significantly higher in age2 samples than that in age29 samples (*p* ≤ 0.05). The relative abundance of *Chaetomium, Fusicolla*, and *Chrysosporium* was significantly higher in age19 samples than that in age29 samples (*p* ≤ 0.05), while the relative abundance of *Pyrenochaetopsis* and *Phaeosphaeriopsis* was significantly higher in age19 samples than that in age2 samples (*p* ≤ 0.05). In addition, the relative abundance of *Nigrospora* was higher in age29 samples than that in age19 samples (*p* ≤ 0.05). The relative abundance of *Pyrenochaetopsis, Phaeosphaeria, Hannaella, Coprinopsis*, and *Phaeosphaeriopsis* was significantly higher in age29 samples than that in age2 samples (*p* ≤ 0.05).

The sampling points were classified based on specific soil depths and divided into two layers [the shallow layer (0–2 m) and the deep layer (2–6 m)]. The dominant taxa in the shallow layer of soil surrounding landfill included *Alternaria, Pyrenochaetopsis*, Phaeosphaeriaceae unclassified, *Scedosporium, Neocosmospora, Titaea*, and *Neocucurbitaria*, which exhibited higher relative abundances. In contrast, in the deep layer, *Neodevriesia, Pseudallescheria*, and *Fusariella* were more abundant (Supplementary Figure S7b).

### 3.5 Microbial correlation with landfill waste, soil properties, and the main influencing factors

**Figure 5:**
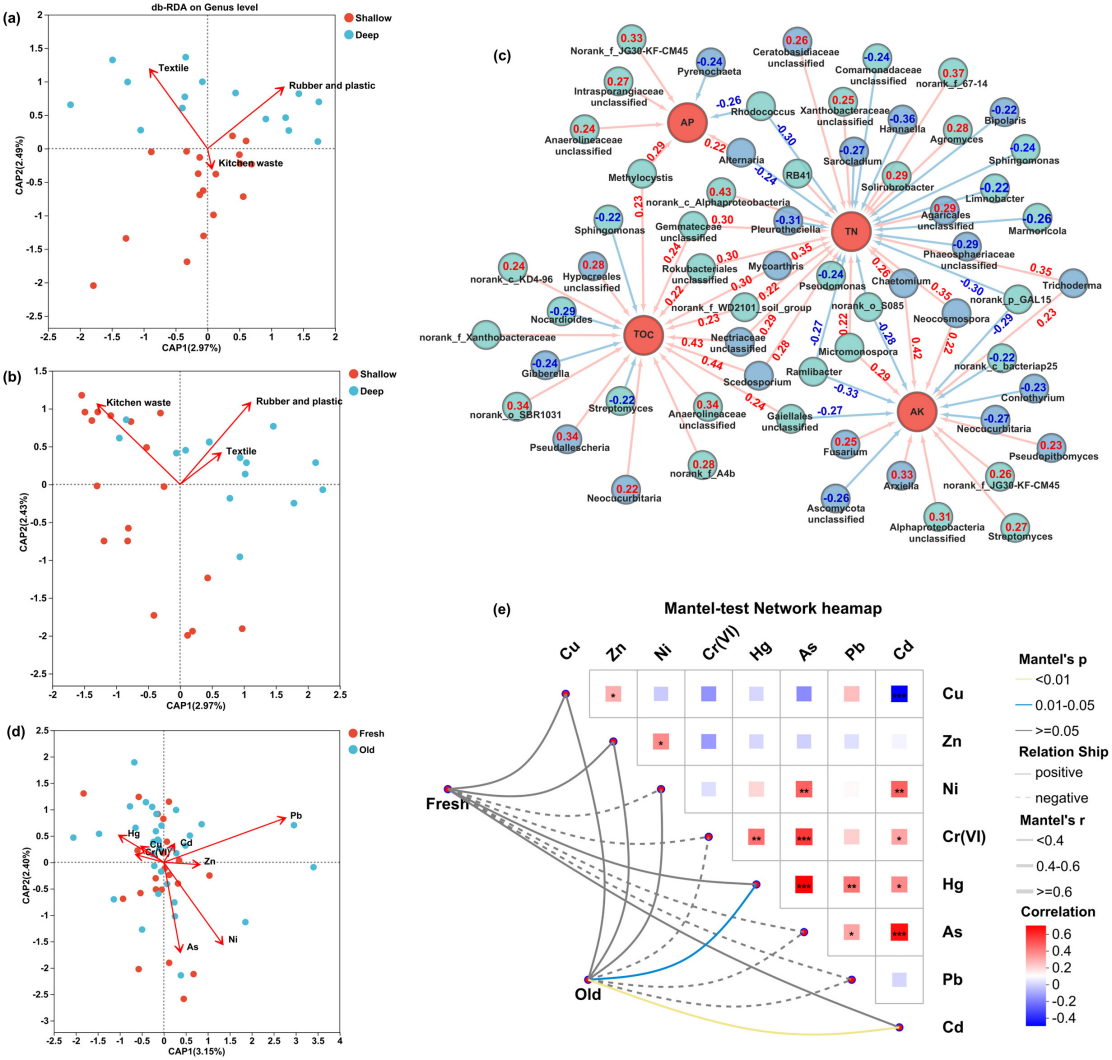
Relationship between soil microbial community and the component of landfill waste, the content of soil nutrients, and heavy metals. **(a)** db-RDA analysis of the major influencing components of landfill waste on the bacterial community in different soil layers. The longer the arrow, the greater the impact of the factor on the microbial community. The distance from the projection point to the origin represented the impact of the factor on the sample microbial community, and the same direction of the point and the arrow indicated a positive correlation. **(b)** db-RDA analysis of important influencing components of landfill waste to fungal community in different soil layers, **(c)** Spearman's correlation analysis between microbial relative abundance and soil nutritional content (*p* < 0.05), the red rows represented positive correlations, the blue rows represented negative correlations, the values presented the correlation coefficients, **(d)** analysis of important influencing elements of heavy metals to soil bacterial community across landfill age gradients by db-RDA, **(e)** the Mantel test examining critical influencing elements of heavy metals to soil fungal community across landfill age gradients. The yellow and blue lines indicated significant correlation between fungal community with related factors (*p* < 0.05).

To investigate the correlation between the composition of soil microbial community and the composition of landfill waste, soil nutrient contents (TN, AP, AK, and TOC), and heavy metal elements [Cu, Zn, Ni, Cr (VI), Hg, As, Pb, and Cd], the db-RDA, Mantel test and Spearman's correlation analysis were performed. The db-RDA analysis revealed that the influence of waste composition on microbial diversity varied with soil depth. The microbial diversity in shallow soil layers exhibited a significant correlation with kitchen waste (*p* ≤ 0.05), while in deeper soil layers, it was primarily influenced by textile, rubber, and plastic waste (*p* ≤ 0.05; Figures 5a, b; Supplementary Figure S9).

The results revealed significant correlations (*p* ≤ 0.05, n = 59) between soil nutritional indices and microbial community compositions, as shown in Figure 5c. The bacterial communities holding significant positive correlations with TN included norank_c_Alphaproteobacteria, norank_f_67-14, Gemmateceae unclassified, Rokubacteriales unclassified, and *Solirubrobacter* (*p* ≤ 0.05), while the bacterial communities such as *Rhodococcus, Ramlibacter, Marmoricola, Sphingomonas*, and *Pseudomonas* were negatively correlated (*p* ≤ 0.05). The norank_f_JG30-KF-CM45, *Methylocystis*, Intrasporangiaceae unclassified, and Anaerolineaceae unclassified were significantly positively correlated with AP (*p* ≤ 0.05), while *Rhodococcus* exhibited maximal negative coefficient (*p* ≤ 0.05). The enrichment of AK positively correlated with communities included Alphaproteobacteria unclassified, *Micromonospora, Streptomyces*, and norank_f_JG30-KF-CM45 (*p* ≤ 0.05), while the enrichment of AK negatively correlated with communities included *Ramlibacter*, norank_p_GAL15, norank_o_S085, Gaiellales unclassified, and norank_c_bacteriap25 (*p* ≤ 0.05). Additionally, taxes such as Anaerolineaceae unclassified, Gaiellales unclassified, Gemmateceae unclassified, *Methylocystis*, and Rokubacteriales unclassified were significantly positively correlated with TOC (*p* ≤ 0.05), while *Nocardioides, Streptomyces*, and *Sphingomonas* were significantly negatively correlated (*p* ≤ 0.05).

Soil fungi communities that positively correlated with TN included *Neocosmospora, Trichoderma, Mycoarthris, Scedosporium*, and *Chaetomium* (*p* ≤ 0.05). The communities with negative correlation were *Hannaella, Pleurotheciella, Sarocladium, Alternaria*, and *Bipolaris* (*p* ≤ 0.05). The community *Alternaria* had maximal positive coefficient with AP (*p* ≤ 0.05), while the community *Pyrenochaeta* was negatively correlated (*p* ≤ 0.05). The fungi that positively correlated with AK included *Chaetomium, Arxiella, Pseudopithomyces, Fusarium*, and *Neocosmospora* (*p* ≤ 0.05), while *Neocucurbitaria*, Ascomycota unclassified, and *Coniothyrium* were negatively correlated (*p* ≤ 0.05). *Scedosporium*, Nectriaceae unclassified, *Pseudallescheria, Mycoarthris*, and *Neocucurbitaria* were significantly positively correlated with TOC (*p* ≤ 0.05), while *Gibberella* was negatively correlated (*p* ≤ 0.05).

The results of db-RDA analysis demonstrated that the influence of heavy metal elements on soil bacteria communities in the soil surrounding the landfill was Pb > Ni > As > Hg > Zn > Cr(VI) > Cu > Cd, and the results of Mantel test analysis demonstrated that Hg and Cd had a significant impact on the fungal diversity of the soil surrounding the landfill (*p* ≤ 0.05; Figures 5d, e). The results of Spearman's correlation analysis indicated that the dominant bacterial and fungal composition in the soil was significantly correlated with the heavy metal elements in the soil environment (*p* ≤ 0.05). The bacterial genera *Agromyces, Streptomyces*, and *Pedomicrobium* were significantly positively correlated with Ni (*p* ≤ 0.05), the genera Vicinamibacteraceae norank, Gemmatimonadaceae norank, JG30-KF-CM45 norank, and *Streptomyces* were significantly positively correlated with As (*p* ≤ 0.05), the genus *Lysobacter* was significantly positively correlated with Hg (*p* ≤ 0.05), the genera *Sphingomonas* and Intrasporangiaceae unclassified were significantly positively correlated with Cu (*p* ≤ 0.05), and the genus *Streptomyces* were significantly positively correlated with Cd (*p* ≤ 0.05). The fungal genera *Neocosmospora, Mycoarthris, Chaetomium, Cephalotrichum, Stilbella*, and *Pseudogymnoascus* were positively correlated with Cr(VI), Hg, As, and Pb, with some correlation coefficients reaching statistical significance (*p* ≤ 0.05). The *Neocosmospora, Mycoarthris, Chaetomium, Stilbella*, and *Pseudogymnoascus* were significantly positively correlated with Cd (*p* ≤ 0.05), *Mortierella, Mycoarthris, Chaetomium, Cephalotrichum*, and *Pseudogymnoascus* were significantly positively correlated with Ni (*p* ≤ 0.05), *Gibberella, Pseudopithomyces, Hannaella, Sarocladium*, and *Periconia* were significantly positively correlated with Cu (*p* ≤ 0.05), and *Pyrenochaetopsis, Hannaella*, and *Sarocladium* were significantly positively correlated with Zn (*p* ≤ 0.05; Supplementary Figure S10).

**Figure 6:**
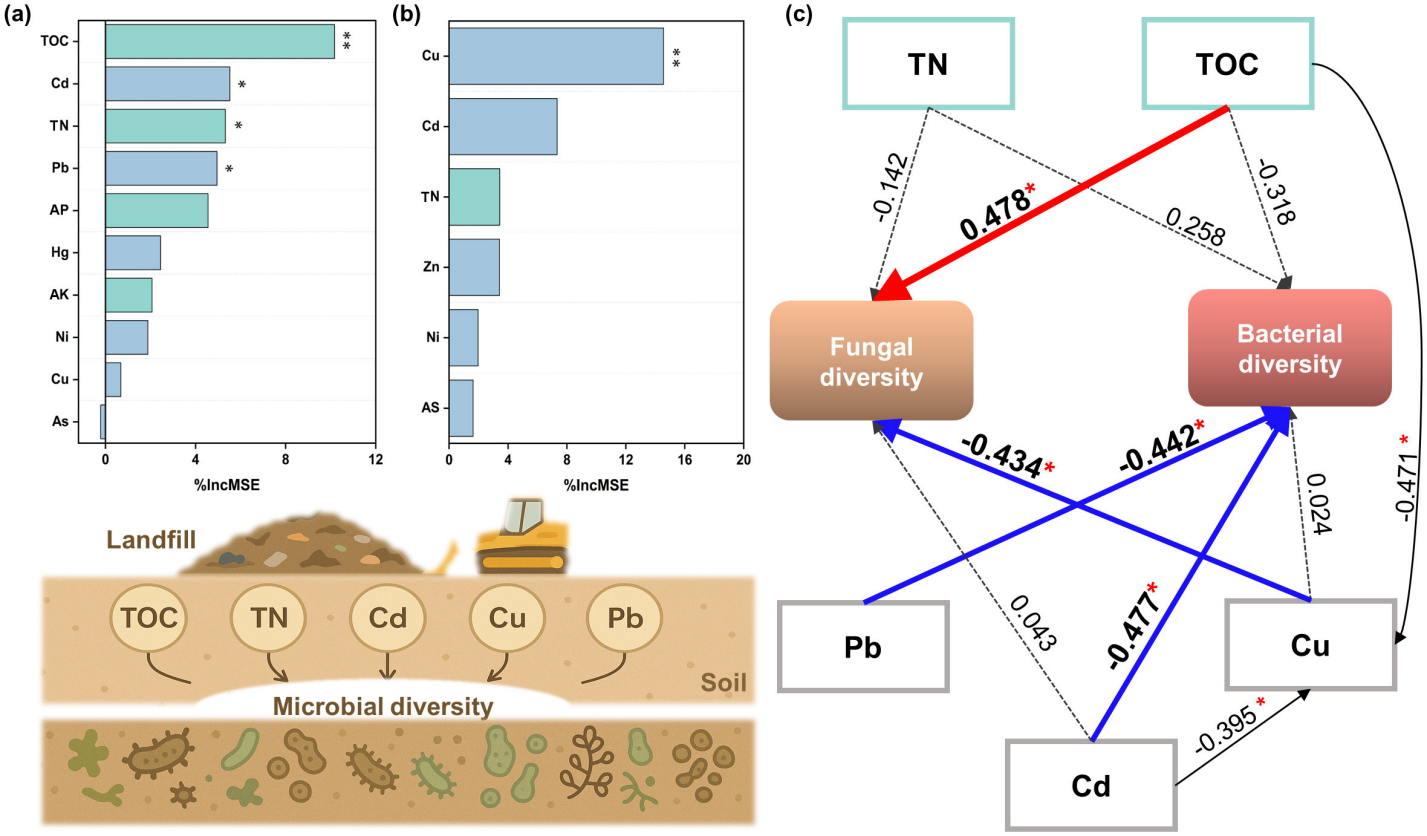
Potential driving mechanisms of soil properties on microbial community structure. Random forest predicted the importance of soil nutrient characteristics and soil heavy metal content to **(a)** bacterial diversity, **(b)** fungal diversity (^*^P < 0.05, ^**^P < 0.01), **(c)** the structural equation model (SEM) described the main pathways by which soil factors affected microbial diversity. The solid line indicated a significant correlation (*p* < 0.05, the red indicated a positive correlation between the variables and microbial diversity, while the blue indicated a negative correlation), and the dotted line indicated an insignificant correlation.

The random forest and structural equation models were further established to evaluate the relative importance of the filtered soil indices and bacterial diversity and fungal diversity (the potential explanatory variables exhibiting VIF > 10 and weak correlation were excluded from subsequent analysis, Supplementary Figure S11). As shown in Figure 6, the soil TOC and heavy metals including Cu, Cd, and Pb had direct correlations with the microbial diversity (*p* ≤ 0.05). TOC was a vital predictor of fungal diversity and had a significantly positive effect on fungal diversity (*p* ≤ 0.05). Soil heavy metal Cu was significantly negatively correlated with fungal diversity, while Cd and Pb were significantly negatively correlated with bacterial diversity (*p* ≤ 0.05), which indicated the negative effects of soil heavy metals on microbial diversity.

### 3.6 Microbial functional prediction of soil nutrient availability

According to the functional prediction of PICRUSt2, the functional genes of bacteria in all soil samples were primarily metabolic pathways, biosynthesis of secondary metabolites, and microbial metabolism in diverse environments (Supplementary Figure S12), indicating that the microbial metabolism in the soil was vigorous and had strong adaptability to complex environments. Consistent with the enrichment trend of soil TN, AP, and AK at depths above 2 m surrounding aged landfill area, the results demonstrated an increase in bacterial genes related to oxidoreductase with proton synthesis and transfer (EC 1.1.1.100, EC 1.2.7.3, EC 1.6.5.3), as well as an increase in bacterial genes related to amidotransferase (EC 6.3.5.6, EC 6.3.5.7) with phosphate release of *Methylocystis*, norank_f_Anaerolineaceae, and Intrasporangiaceae unclassified. Fungal genes related to protein phosphotransferases (EC 2.7.1.37), cellobiose (EC 3.2.1.21), and α-glucanase (EC 3.2.1.3) and some monooxygenase (EC 1.14.14.1, EC 1.14.13.1) of *Alternaria, Chaetomium, Pyrenochaeta, Neocosmospora, Scedosporium*, and *Mycoarthris* were also increased (Supplementary Figure S13). In the soil layer below 2 m surrounding the aged landfill area, TN and AP showed a negative correlation with *Rhodococcus* and *Ramlibacter*, which held increasing genes related to acyl-CoA dehydrogenase (EC 1.3.8.7), hydrase (EC 4.2.1.17), glutarate-semialdehyde dehydrogenase (EC 1.2.1.20), and succinate-semialdehyde dehydrogenase (EC 1.2.1.16, EC 1.2.1.79; Figure 6). *Mycoarthris* and *Pseudallescheria* were consistently enriched with TOC content in soil surrounding the fresh landfill area than the aged landfill. Simultaneously, fungal genes related to phosphotyrosine phosphatase (EC 3.1.3.16), alkaline protease (EC 3.4.25.1), alkyl/aryl transferase (EC 2.5.1.18), and glycosylases (EC 3.2.1.21, EC 3.2.1.58) increased (Figure 6). Therefore, soil nutrient distribution surrounding the landfill was affected by the structure and function of the soil microbial community.

## 4 Discussion

### 4.1 Characteristics of soil microbial structure around landfill

In fact, numerous studies have detected widespread dominant microorganisms in the air and rivers near the landfill (Abiriga et al., 2021). However, research on the surrounding soil of landfills has been relatively limited. Reports have shown that the total OTU number of bacterial OTUs both domestically and internationally rarely exceeds 4,000 (Stamps et al., 2016), which is lower than the bacterial diversity observed in this study. Studies on fungal diversity in landfills are scarce. For instance, a report indicated that the fungal Shannon index and Chao1 index in the solid waste and leachate samples from a closed landfill were ~2.7 and 250, respectively (Ye et al., 2021), both of which were lower than the soil fungal indices value surrounding the investigated landfill. Therefore, the landfill has not caused a drastic disturbance to the microbial diversity of the surrounding soil. The Shannon and Chao1 indices values of soil microorganisms at 30–45% points surrounding the landfill are higher than those at the control points, indicating that the microbial diversity is more abundant in the soil surrounding the landfill.

A substantial portion of bacterial populations in the soil surrounding the landfill are uncultivable, comprising over 60% of the total bacterial community, indicating the complexity of the bacterial community structure in this study. All samples, including those of the control points, have no unique bacterial OTU. Compared with the control points in soil around the landfills, at the phylum level, the relative abundance of Proteobacteria, Actinobacteriota, and Bacteroidota increased, while that of Chloroflexi, Acidobacteriota, and Planctomycetota decreased, presenting the bacterial composition characteristics of landfills (Wang et al., 2021). At the genus level, the relative abundance of *Vicinamibacterales* norank decreased, while that of *Pseudomonas, Nocardioides, Comamonadaceae, Marmoricola, Rhodococcus*, and *Lysobacter* increased. These genera are dominant bacterial taxa commonly associated with landfills. Specifically, *Pseudomonas, Nocardioides, Marmoricola*, and *Rhodococcus* have been previously annotated as dominant bacteria in landfills (Stamps et al., 2016; Selvarajan et al., 2022), and *Comamonadaceae* and *Lysobacter* are commonly identified in the soil and water systems surrounding landfills (De Mandal et al., 2019; Wang et al., 2022). This finding indicated that the landfills in this study area primarily affect the relative abundance of bacteria in the surrounding soil.

Compared with control points, in the soil surrounding landfills, *Blastocladiomycota, Humicola, Ganoderma, Emericellopsis, Calceomyces, Purpureocillium*, and *Paraphysoderma were* significantly decreased (*p* ≤ 0.05), which were reported to be sensitive to the environment and less competitive (Kepenekci et al., 2018; Passarini et al., 2022). However, the relative abundance of *Alternaria, Pyrenochaetopsis*, and *Fusarium* increased. *Fusarium* was the dominant genus commonly found in landfills (Ye R. et al., 2024), and *Alternaria* has also been reported to be widely present in bioaerosols above landfills (Fraczek et al., 2017). In addition, several unique fungal genera were identified in the soil surrounding landfills, including *Arxiella, Pseudopithomyces, Neodevriesia, Subulicystidium, Pleurotheciella, Phaeosphaeria, Coniothyrium, Fusariella, Neocucurbitaria*, and *Bipolaris*, which have been commonly identified in domestic waste plastic (Gkoutselis et al., 2021; Rüthi et al., 2023), asbestos fiber (Berry et al., 2024), crop residues (Das and Abdulhameed, 2020), and other kinds of wastes (Chigwada et al., 2023; Liu et al., 2024). Therefore, landfills not only influence the abundance of fungal communities in the surrounding soil but also shape a region-specific population composition, thereby affecting the overall fungal microbial structure.

### 4.2 Influence of soil environmental factors on the microbial community and its spatio-temporal distribution characteristics

The response analysis results between soil microbial diversity and potential explanatory factors demonstrated that soil TOC was an important factor affecting microbial diversity. Compared with bacteria, TOC exhibited a positive effect on fungal diversity, which may result from the finding that fungi had a higher demand for carbon resources and required a higher and wider C: N ratio than the bacteria (Zang et al., 2024). This study demonstrated that soil heavy metal Cu had a significant negative impact on fungal diversity, while Cd and Pb had a significant negative impact on bacterial diversity. It is worth noting that there is a certain variability in the effects of heavy metals on microbial diversity and their significance in different surveys (Yao et al., 2022; Zhou et al., 2024), which is essentially due to differences in the tolerance of different taxa in the microbial community to heavy metal stress (Campillo-Cora et al., 2025). This study further explored the association between microbial composition and the presence of heavy metals.

Landfill-specific soil bacteria mentioned above, including *Pseudomonas, Nocardioides, Marmoricola*, and *Lysobacter*, were primarily enriched surrounding the aged landfill area, while *Rhodococcus* was abundant in the area surrounding fresh landfill. A recent report demonstrates that these species have strong environmental resistance and the ability to degrade environmental pollutants (Totubaeva et al., 2019). *Pseudomonas* performs organic matter degradation and denitrification, making it an important and flexible metabolically microorganism in soil (Wang et al., 2017). This study found that Lysobacter and *Nocardioides* have the potential to resist exposure to heavy metals such as Hg and Cu in the landfill surrounding soil, and other studies have also reported that these species and *Marmoricola* have strong heavy metal tolerance (Selvarajan et al., 2022; Yang et al., 2024) and exhibit strong degradation ability for refractory organic matter (De Mandal et al., 2019). *Pseudomonas* was primarily enriched in the shallow layer (0–0.5m), where the accumulation of exogenous organic matter in the surface soil, such as animal and plant, remains, and drips during leachate transportation might provide a variety of substrates, including amino acids, sugars, aromatic hydrocarbons, and organic acids (Wang et al., 2021). *Marmoricola, Nocardioides*, and *Rhodococcus* were primarily enriched in the deep layer below 2 m. Both *Nocardioides* and *Rhodococcus* belong to the family Nocardiaceae. A recent report demonstrated that *Nocardioides* is a species that has existed for over 100 million years and can degrade stable organic preservatives and insecticides, such as hexachlorobenzene and pentachlorophenol (Selvarajan et al., 2022). *Rhodococcus* is even more versatile in degrading a wide range of compounds, including both organic and inorganic compounds, and exhibits strong environmental tolerance and persistence (Kim et al., 2018). In this study, it had positive correlations with Zn and Pb, which showed good tolerance to those heavy metals.

The landfill-specific *Alternaria* belongs to the typical filamentous fungi, which can produce spores, and has higher activity in the soil surface layer. In addition, it is adaptable to the environment and is less affected by waste pollutants, exhibiting a positive correlation with kitchen waste, paper, textile, rubber, and plastic of landfill components in this study. Other studies have also reported that *Alternaria* can not only degrade plant lignocellulose but also degrade plastic and has higher abundance in the shallow soil environment surrounding the aged landfill area (Sekhohola-Dlamini and Tekere, 2020). In addition, the dominant genus *Pseudopithomyces* had a strong tolerance to Cu in soil surrounding aged landfill area, which had reported potential tolerance to Cu-enriched environments (Ke et al., 2023), and as a kind of saprophytic fungi, it might reduce Cu content by biological absorption or precipitation (Roy et al., 2025). *Neodevriesia* usually decomposes lignin and cellulose in plant litter or crop residues and tends to be accumulated around fresh landfill area. *Neocucurbitaria* was abundant surrounding the fresh landfill area but nearly absent around the aged area, which was negatively correlated with many heavy metal elements such as Hg, As, and Cd, and its poor adaptability to heavy metal accumulation has been also reported (Ważny et al., 2021).

According to the test assessing the differences between different sampling groups, significant differences in the structure of bacterial and fungal communities were observed in the soil surrounding the landfill at different ages (*p* ≤ 0.05), indicating microbial population succession. *Lacunisphaera* was significantly enriched in the soil surrounding the fresh landfill area (*p* ≤ 0.05), which was a typical cellulose-dependent bacteria in the Firmicutes phylum (Hoang et al., 2024). In addition, Saccharomycetales unclassified and *Chaetomium*, which belong to the Ascomycota phylum (Passarini et al., 2022), were more abundant and were an important phylum that degraded solid waste. This study also found that *Chaetomium* has tolerance to heavy metals (Ni, Hg, As, Pb, and Cd) that enriched in soil surrounding landfills, and its adsorption functions for Pb and Cd have been reported in other studies (Albert et al., 2020; Geetha et al., 2021). As the landfill ages and easily degradable organic matter was consumed, the abundance of autotrophic nitrogen-fixing *Phyllobacterium* and *Allorhizobium* (Ahmad et al., 2012; Sekhohola-Dlamini et al., 2021) as well as *Agromyces, Phaeosphaeriopsis, and Nigrospora* for decomposition of refractory organic matter was increased in age19 and age29 samples. Among them, *Agromyces* was also found to have strong tolerance to heavy metal Ni in this study. It has been considered an important member of the microbial remediation of heavy metal pollution and could maintain high activity under different heavy metal pollution conditions (Wang et al., 2023). The soil microbial community structure had already shown characteristics affected by aged waste pile, when the landfill was 19 years old. Mineralizing *Levilinea, Anaerolinea, Methylosinus, Ellin6067, and* Burkholderiales norank (Feng et al., 2019; Ye J. et al., 2024) and plastic-degrading *Hannaella, Pyrenochaetopsis*, and *Phaeosphaeria* (Duan et al., 2021; Kim et al., 2022) were significantly enriched in age29 samples (*p* ≤ 0.05).

### 4.3 Soil microbial community correlation with major soil nutritional distribution and its functional prediction

In this study, the surrounding soil of landfill sites was primarily used as forest land. Soil TN, AP, AK, and TOC contents of the control points were similar to the official soil survey data of the area (Dai et al., 2018). Compared with the control samples, the TN and TOC contents decreased, while the AP and AK contents increased in soil surrounding the landfill. Although there was almost no risk of leachate leakage from sanitary landfills, the landfill still had an impact on the physical and chemical properties of the surrounding soil. The correlation analysis results between nutritional indices and microorganisms demonstrated that potential organic degrading and denitrifying bacteria such as *Pseudomonas, Marmoricola, Rhodococcus, Sphingomonas*, and *Nocardioides* and fugal genera *Alternaria* and *Gibberella* were significantly negatively correlated with soil TN and TOC contents (*p* ≤ 0.05). Most of the taxa are specific to landfill environments. The bacterial genera norank_f_Anaerolineaceae, unclassified_f_Intrasporangiaceae, and norank_f_JG30-KF-CM45 and fungal genera *Alternaria, Chaetomium, Neocosmospora*, and *Fusarium* were significantly positively correlated with soil AP and AK contents (*p* ≤ 0.05). Other studies have also reported positive correlation between norank_f_JG30-KF-CM45 and *Fusarium* with soil AK content (Maina et al., 2009; Fazl et al., 2024). *Chaetomium* is an important fungus that releases AP and AK into the soil (Zhang et al., 2023). The landfill-specific *Alternaria* is also a typical saprotrophic fungus with P release function (Ceci et al., 2018). Therefore, the diversity of microorganisms at landfill is an important factor influencing changes in the nutrient content of surrounding soils.

According to PICRUSt2 analysis, consistent with the enrichment trend of soil TOC, TN, AP, and AK, the microbial functional genes related to phosphohydrolase and phosphotransferase for P release, as well as genes involved in organic acid synthesis and proton supply and transfer that promote K dissolution (Pandey et al., 2020; Bright et al., 2022), were in abundance in the soil layer above 2 m depth surrounding aged landfill area. However, genes related to fatty acid and amino acid metabolism and carbon fixation (through the 3-hydroxypropio, nate cycle/4-hydroxybutyrate cycle pathway) were more abundant in soil layer below 2 m depth, which could cause the consumption of N and was not conducive to K release. Soil microorganisms in shallow layer mainly decomposed or transformed the refractory organic matter, while vigorous microbial carbon fixation-related metabolism occurred in deep layer, resulting in a large concentration range of soil TOC at different depths. The higher TOC concentration surrounding fresh landfill area might be due to the influx of exogenous organic matter into the soil caused by landfill operations (Lemanowicz et al., 2016). Microorganisms that were positively correlated with these nutrients possessed a rich set of genes related to organic substrate hydrolyzing enzymes and alkyl/aromatic transfer enzymes. Therefore, the soil nutrients surrounding the landfill had complex spatio-temporal distribution characteristics affected by the structure and function of the soil microbial community.

## 5 Conclusion

Based on the above research results, the following conclusions can be drawn: (1) The landfill-specific dominant bacteria such as *Pseudomonas, Marmoricola, Rhodococcus, Sphingomonas*, and *Nocardioides* and fungi such as *Alternaria, Pyrenochaetopsis*, and *Fusarium* were enriched in surrounding soil; (2) the soil dominant microorganisms showed changes in abundance at different landfill ages and soil depths. Bacteria and fungi related to the degradation of organic matter, such as cellulose, were enriched in the soil surrounding fresh landfill area. As landfill age increased, the abundance of autotrophic nitrogen-fixing bacteria and bacteria that decompose refractory organic matter and environmental tolerants increased; (3) the soil TOC and heavy metals including Cu, Cd, and Pb had strong direct correlations with the bacterial and fungal diversity. While heavy metals generally showed negative effects on microbial diversity, several dominant microbial taxa in the soil exhibited potential tolerance to heavy metal stress; (4) in the soil around the landfill, ~1.5–3 times higher concentrations of total nitrogen (TN), available phosphorus (AP), and available potassium (AK) were observed in the upper 2-m layer near the aged landfill areas, whereas higher concentration of total organic carbon (TOC) was detected surrounding the fresh landfill area. The distribution of microbial taxa and predicted functional profiles were strongly associated with the nutrient availability. Therefore, landfill activities have affected the structure and function of the surrounding soil microbial community, causing the content of the main soil nutrients (C, N, P, and K) to exhibit complex spatio-temporal distribution characteristics.

## Data Availability

The datasets presented in this study can be found in online repositories. The names of the repository/repositories and accession number(s) can be found below: https://www.ncbi.nlm.nih.gov/, PRINA1206814.
