# Supplementary material for: Spatio-temporal distribution of soil microbial communities and nutrient availability around a municipal solid waste landfill
Source: Front Microbiol. 2025 Jun 16;16:1583149. doi: 10.3389/fmicb.2025.1583149 (PMC12206801; doi:10.3389/fmicb.2025.1583149)
Supplement: Supplementary file 1 [file Data_Sheet_1.docx]

**Spatio-temporal distribution of soil microbial communities and nutrient availability around a municipal solid waste landfill**

1. **Supplementary methods**
   1. **Sample collection and pretreatment**

This soil sampling used disposable plastic white tube sampler, which is equipped with a booster to push the soil into the sample bottle. After the soil samples were brought back to the laboratory, the samples used for nutritional index measurement were temporarily stored at 4°C, and the samples used for microbial diversity analysis were temporarily stored at -20°C.

The soil samples need to be pretreated before testing. The samples were properly crushed, turned, air-dried, and passed through a 2mm sieve (10 mesh) to remove gravel and plant roots to reduce experimental errors; the sieve-underneath material was crushed again, impurities are picked out, and after mixing, the treated samples could be used after passing through a 0.25mm sieve (60 mesh).

- 1. **DNA quality measurement**

The quality and concentration of DNA were determined by 1.0% agarose gel electrophoresis and a NanoDrop2000 spectrophotometer (Thermo Scientific, United States) and kept at -80℃ prior to further use.

- 1. **PCR amplification**

The hypervariable region V4 of the bacterial 16S rRNA gene were amplified with primer pairs 515F (5'-GTGCCAGCMGCCGCGG-3') and 806R (5'-GGACTACHVGGGTWTCTAAT-3'), while primer pairs ITS1F (5’-CTTGGTCATTTAGAGGAAGTAA-3’) and ITS2R (5’-GCTGCGTTCTTCATCGATGC-3’) were used as forward and reverse primer respectively for fungal PCR amplification byT100 Thermal Cycler PCR thermocycler (BIO-RAD, USA). The PCR reaction mixture including 10 μL 2× Taq Plus Master Mix, 0.8 μL each primer (5 μM), 10 ng of template DNA, and ddH2O to a final volume of 20 µL. PCR amplification cycling conditions were as follows: initial denaturation at 95 ℃ for 5 min, followed by 35 cycles of denaturing at 95 ℃ for 30 s, annealing at 58 ℃(for 16S, and 60℃ for ITS) for 30 s and extension at 72 ℃ for 1 min, and end at 4 ℃.

- 1. **Biological statistical analysis**

Raw FASTQ files were de-multiplexed using an in-house perl script, and then quality-filtered by Fastp version 0.19.6, and merged by Flash version 1.2.11, with the following criteria: (i) the reads were truncated at any site receiving an average quality score of <20 over a 50 bp sliding window, and the truncated reads shorter than 50 bp were discarded, reads containing ambiguous characters were also discarded; (ii) only overlapping sequences longer than 10 bp were assembled according to their overlapped sequence. The maximum mismatch ratio of overlap region is 0.2 Reads that could not be assembled were discarded; (iii) Samples were distinguished according to the barcode and primers, and the sequence direction was adjusted, exact barcode matching, 2 nucleotide mismatches in primer matching.

Such as Chao1 richness and Shannon index are calculated with Mothur v1.30.2[19]. In order to identify the species taxonomic information of each OTU, using the RDP classifier version 2.13(https://sourceforge.net/projects/rdp-classifier/) to perform taxonomic analysis on the OTU representative sequences with 97% similar levels, and determined the community species composition of each sample at each taxonomic level (Domain, Kingdom, Phylum, Class, Order, Family, Genus, Species). Take SILVA version 138 (http://www.arb-silva.de), RDP version 11.5 (http://rdp.cme.msu.edu/) and GreenGenes version 135 (http://greengenes.secondgenome.com/) as the comparison databases of 16s bacterial and archaeal ribosome RNA, while using UNITE version 8.0 (http://unite.ut.ee/index.php) as comparison database of fungal ITS. Functional genes were compared through FunGene version 9.6 (<http://fungene.cme.msu.edu/>). Using Qiime version 1.9.1 (http://qiime.org/scripts/assign_taxonomy.html) and RDP Classifier version 2.13 (http://sourceforge.net/projects/rdp-classifier/) to calculate and output, confidence threshold of 0.7.

The OTU abundance table was standardized with Phylogenetic Investigation of Communities by Reconstruction of Unobserved States 2 (PICRUSt2), a software package for the functional prediction of amplified 16S rRNA sequences, which removes the effect of the copy number of the 16S rRNA gene in the species genome. Each OTU corresponding to a phylogenetic lineage in this software was then used to annotate the Kyoto Encyclopedia of Genes and Genomes (KEGG) to produce the OTU annotation information for each functional level and the abundance information. PICRUSt2 was also used to analyze the fungal ITS sequences.

1. **Supplementary figures**


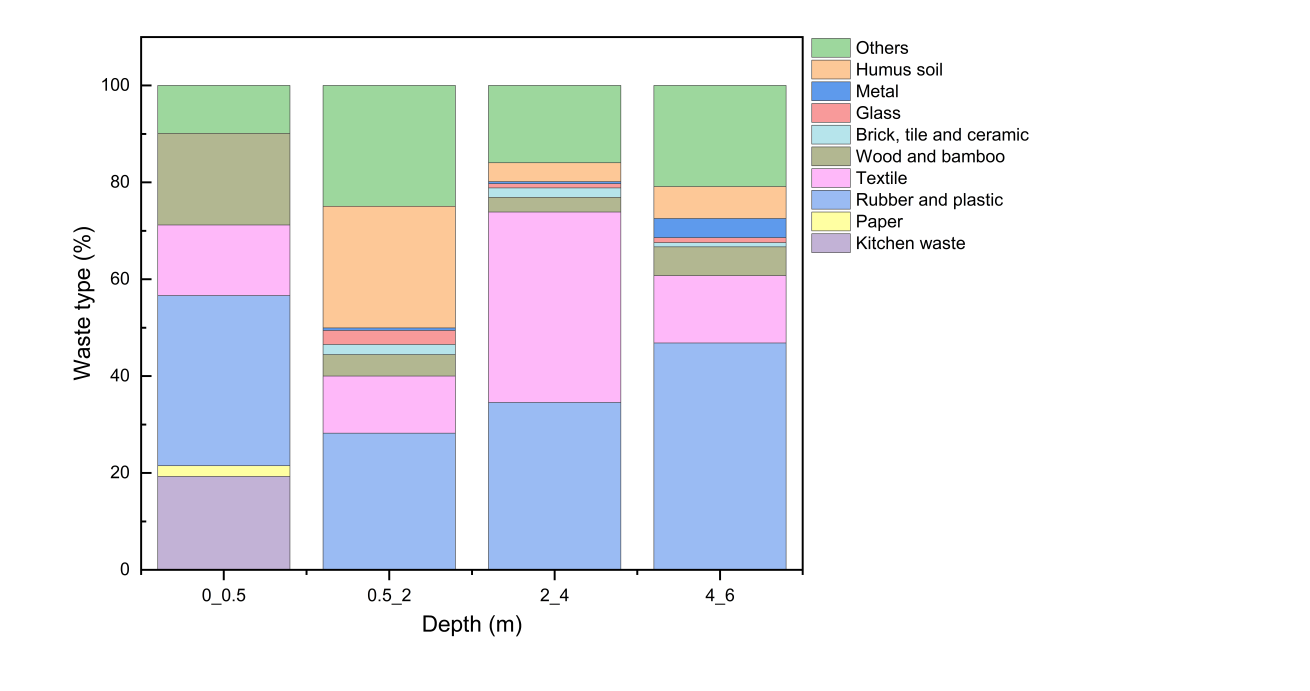


**Figure S1** The component of landfill waste in different depth.

**
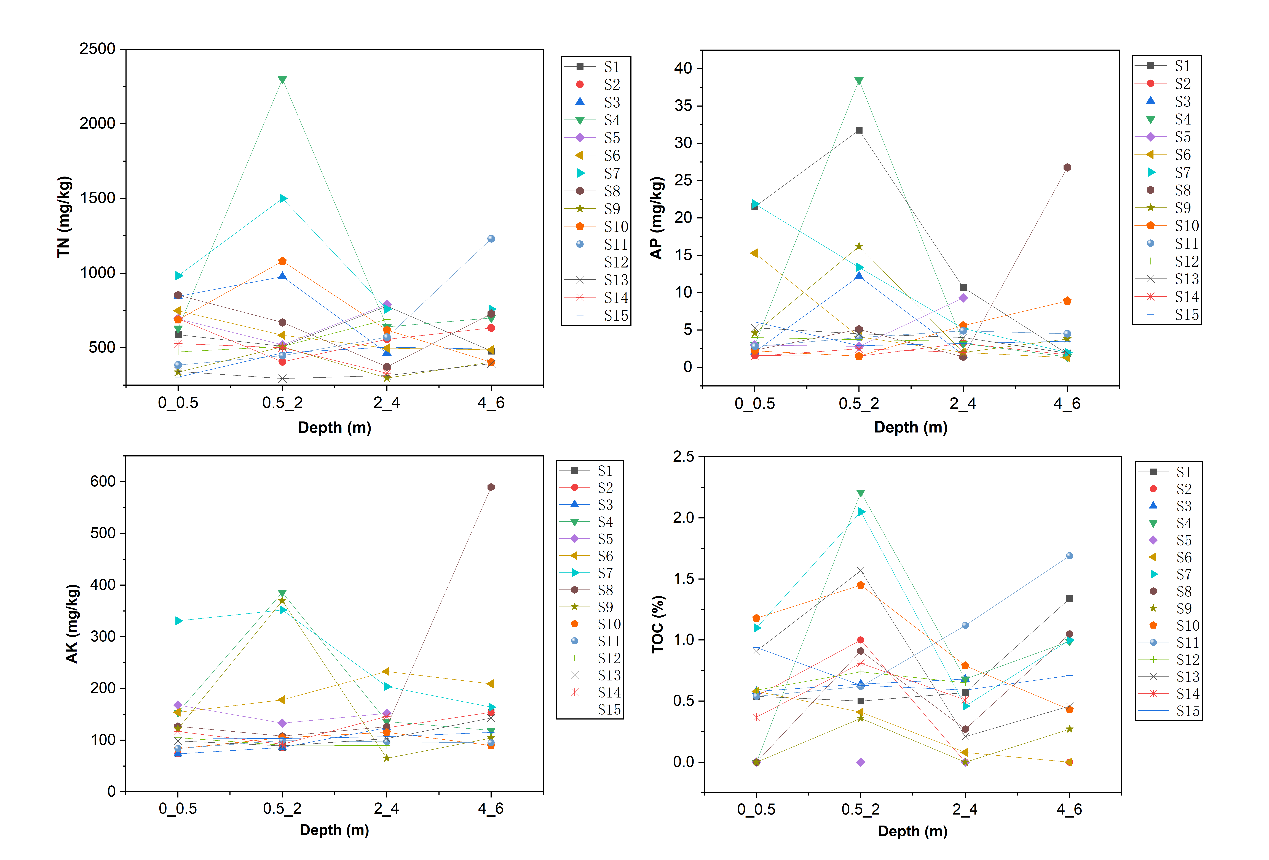
**

**Figure S2** The content of soil nutritional indices in different depth. (a) TN, (b) AP, (c) AK, (d) TOC





**Figure S3** The content of soil heavy metals in different samples.


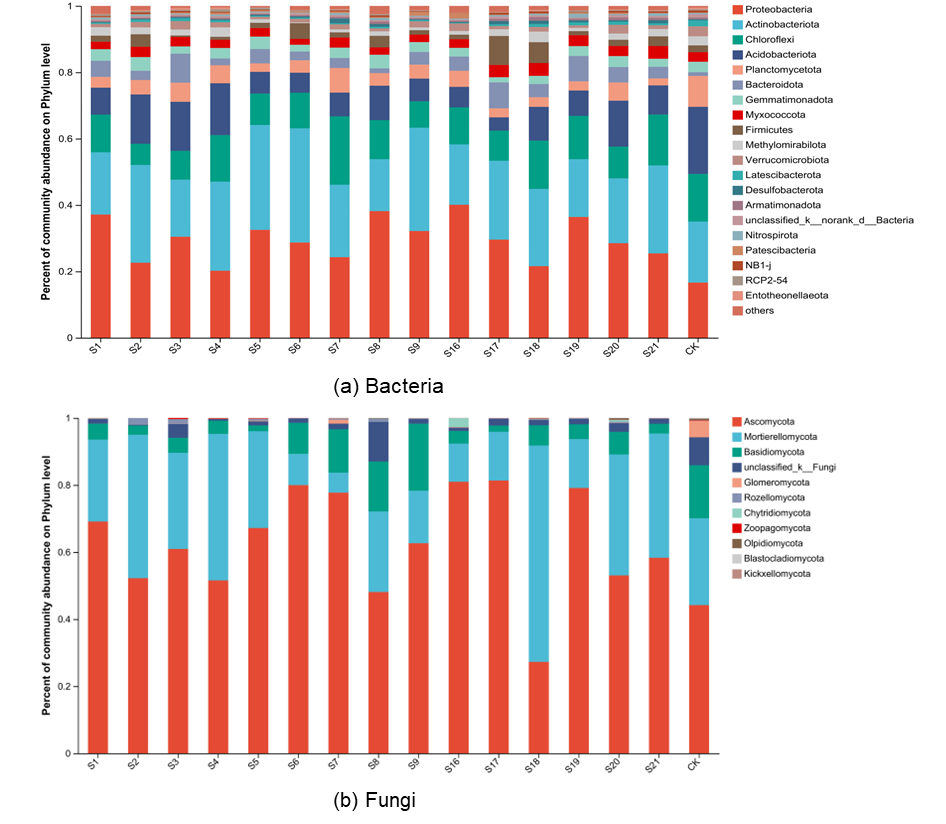


**Figure S4** The dominant bacterial (a) and fungal (b) composition in different samples at phylum level. The relative abundance of each phylum reflects the average level of different depth at the sampling point.

**
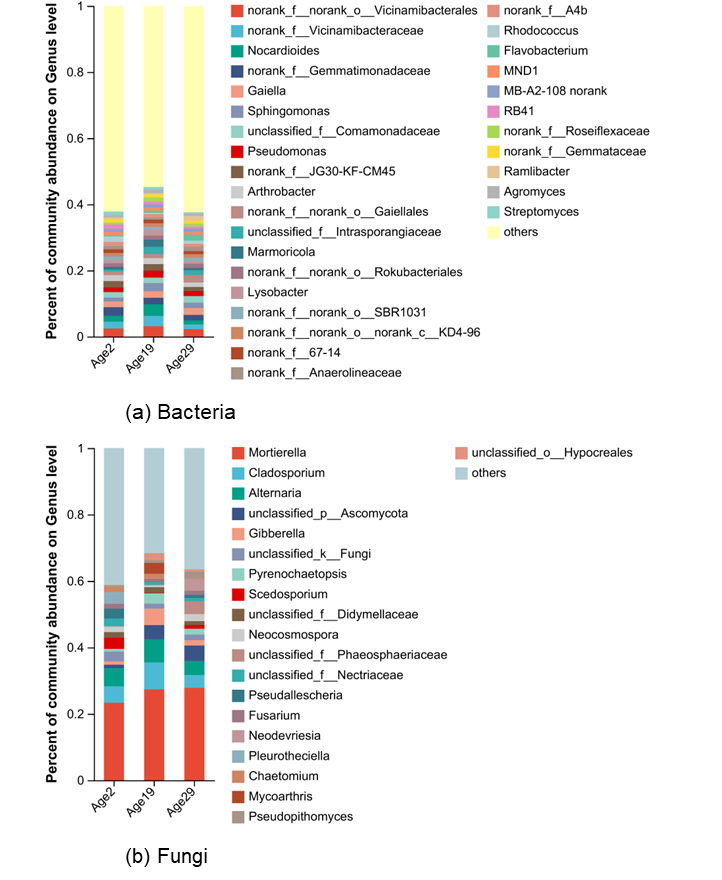
**

**Figure S5** The top 20 dominant bacterial (a) and fungal (b) composition in soil samples around landfill areas of different age at genus level.


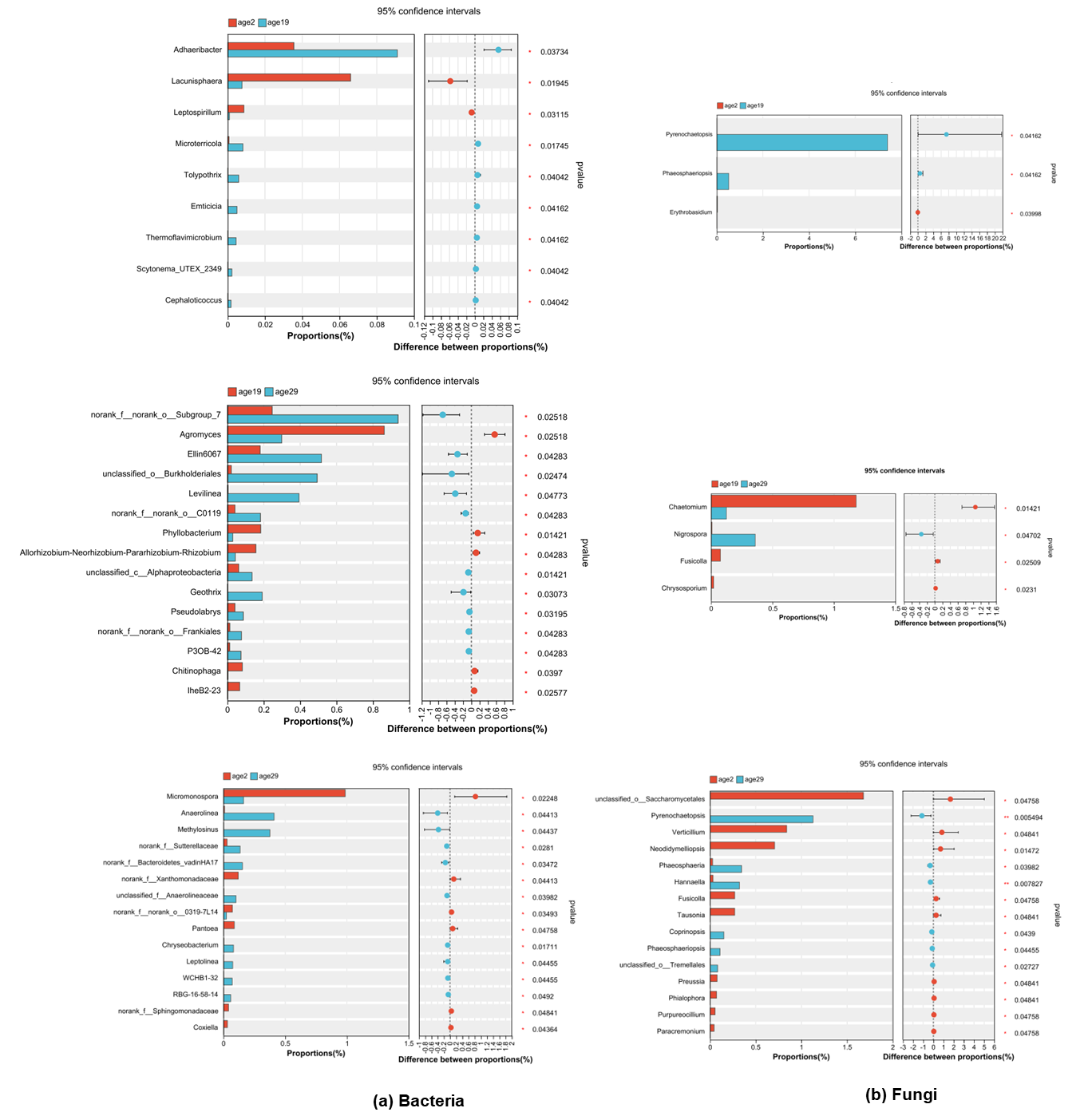


**Figure S6** The Kruskal-Wallis rank sum test assesses the differences between

sampling points around landfills of different age at genus level.


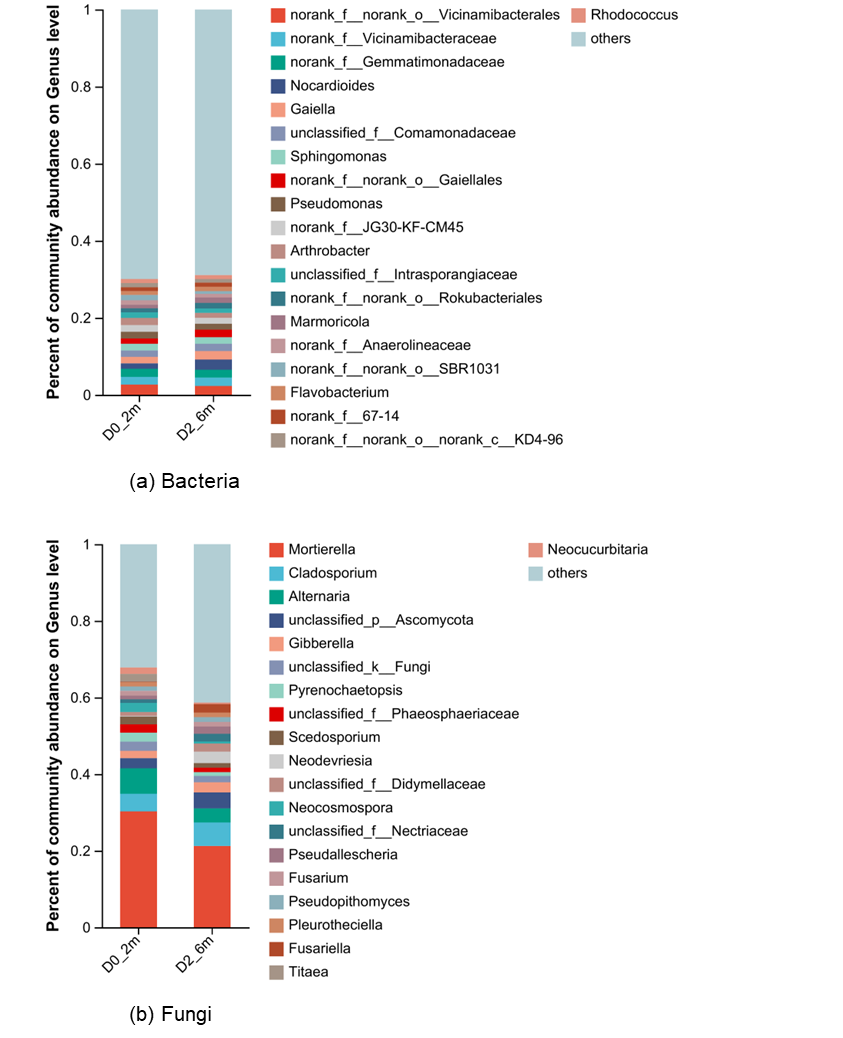


**Figure S7** The top 20 dominant bacterial (a) and fungal (b) composition in soil samples in different depth at genus level.


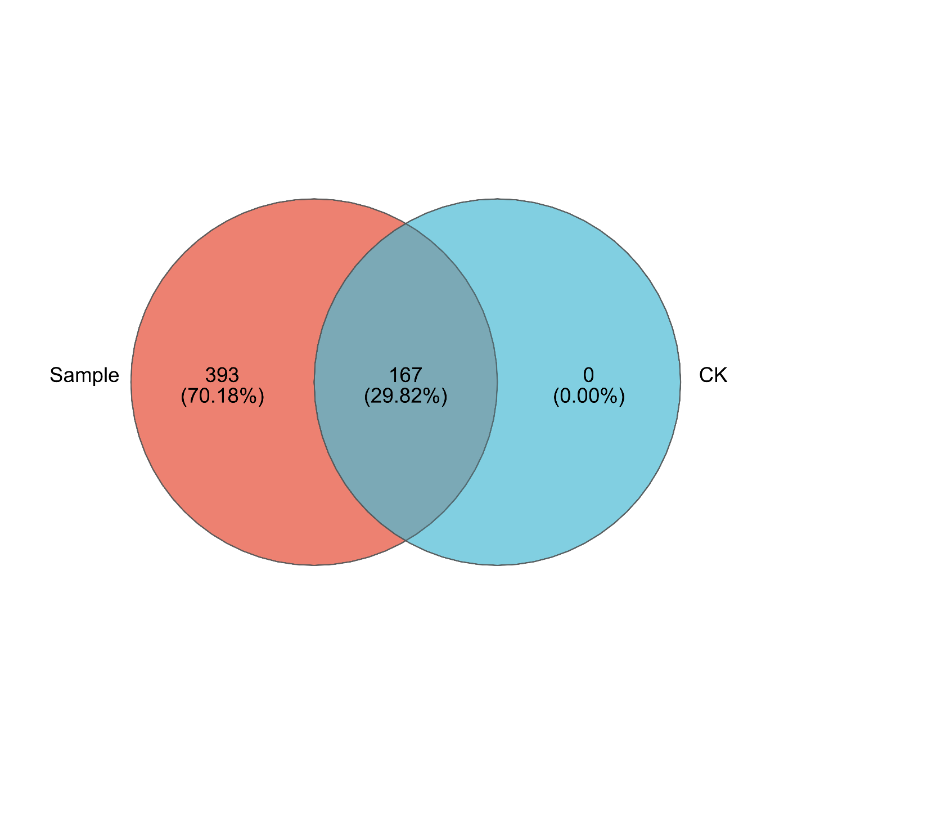


**Figure S8** The unique fungal composition in soil at genus level.


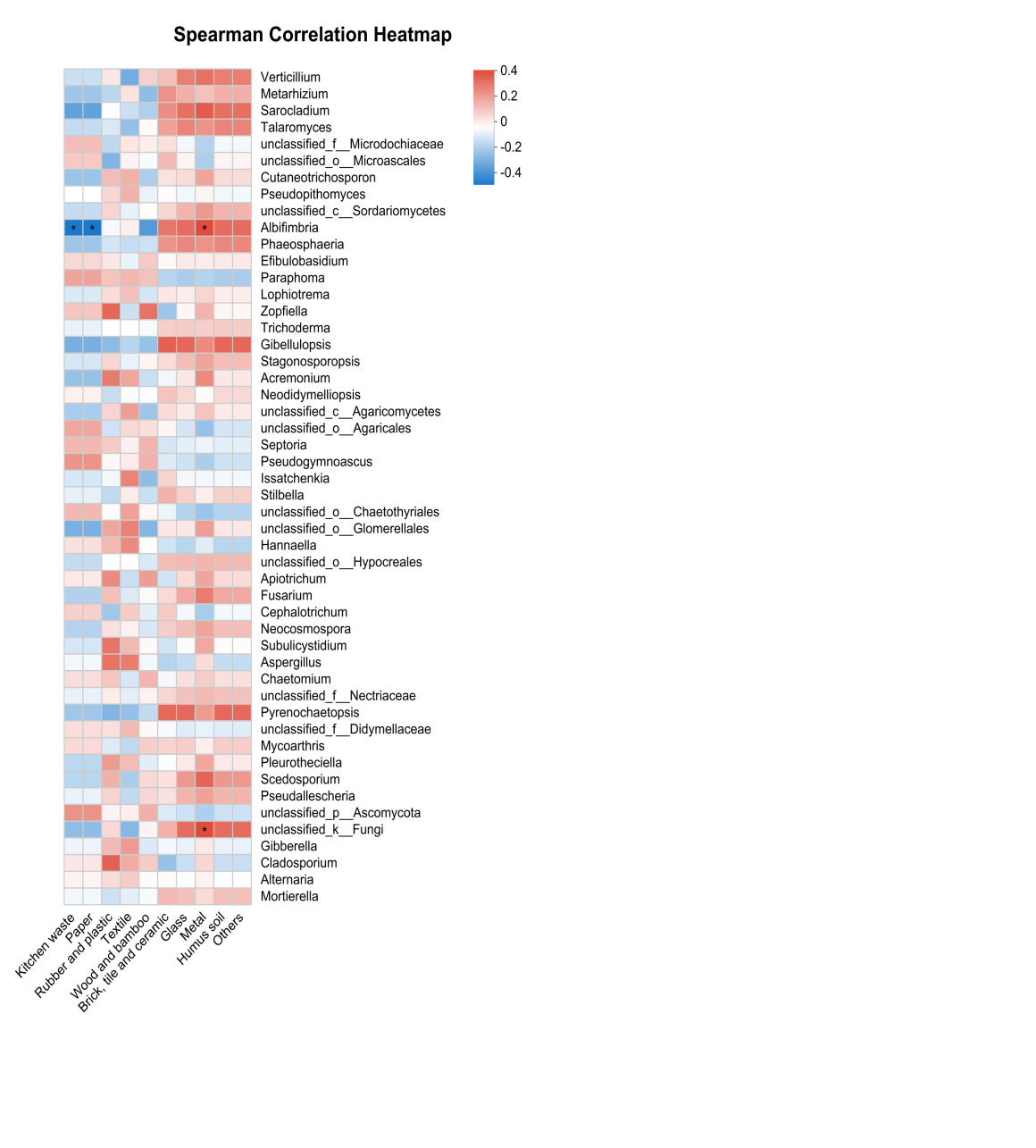

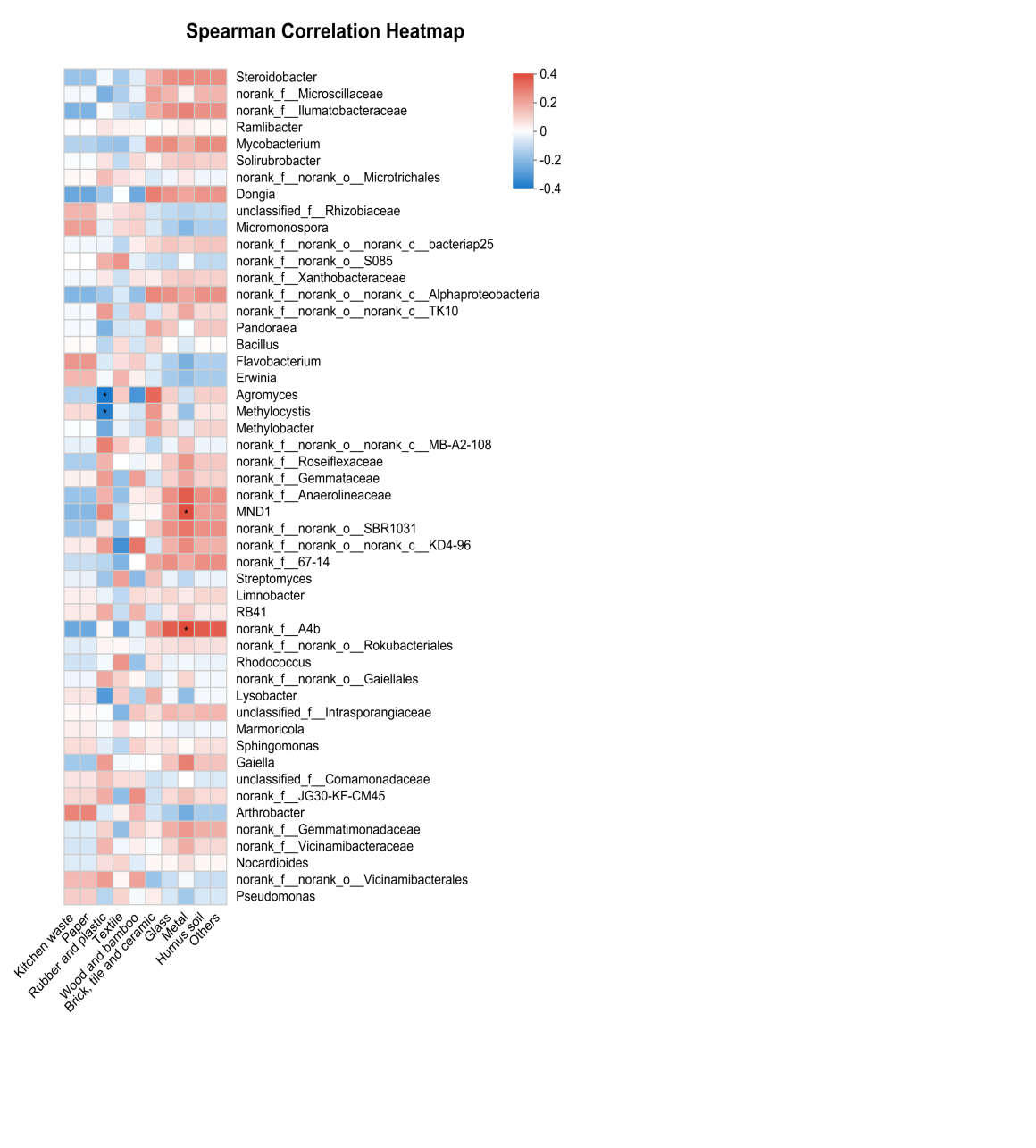


(a)

(b)

**Figure S9** The relationship between soil microbial community and landfill waste by Spearman correlation analysis. (a) was related to bacterial community, (b) was related to fungal community


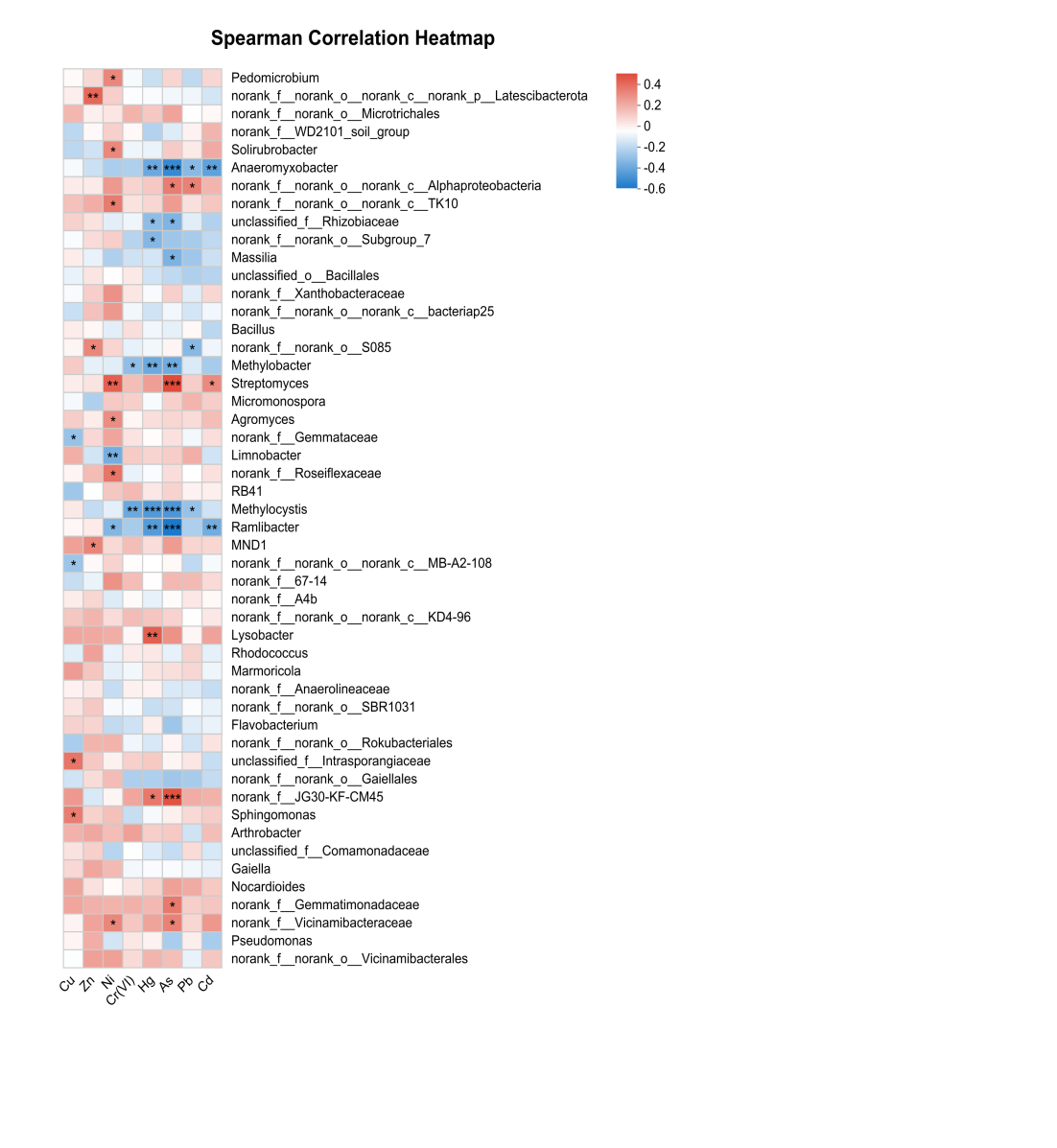

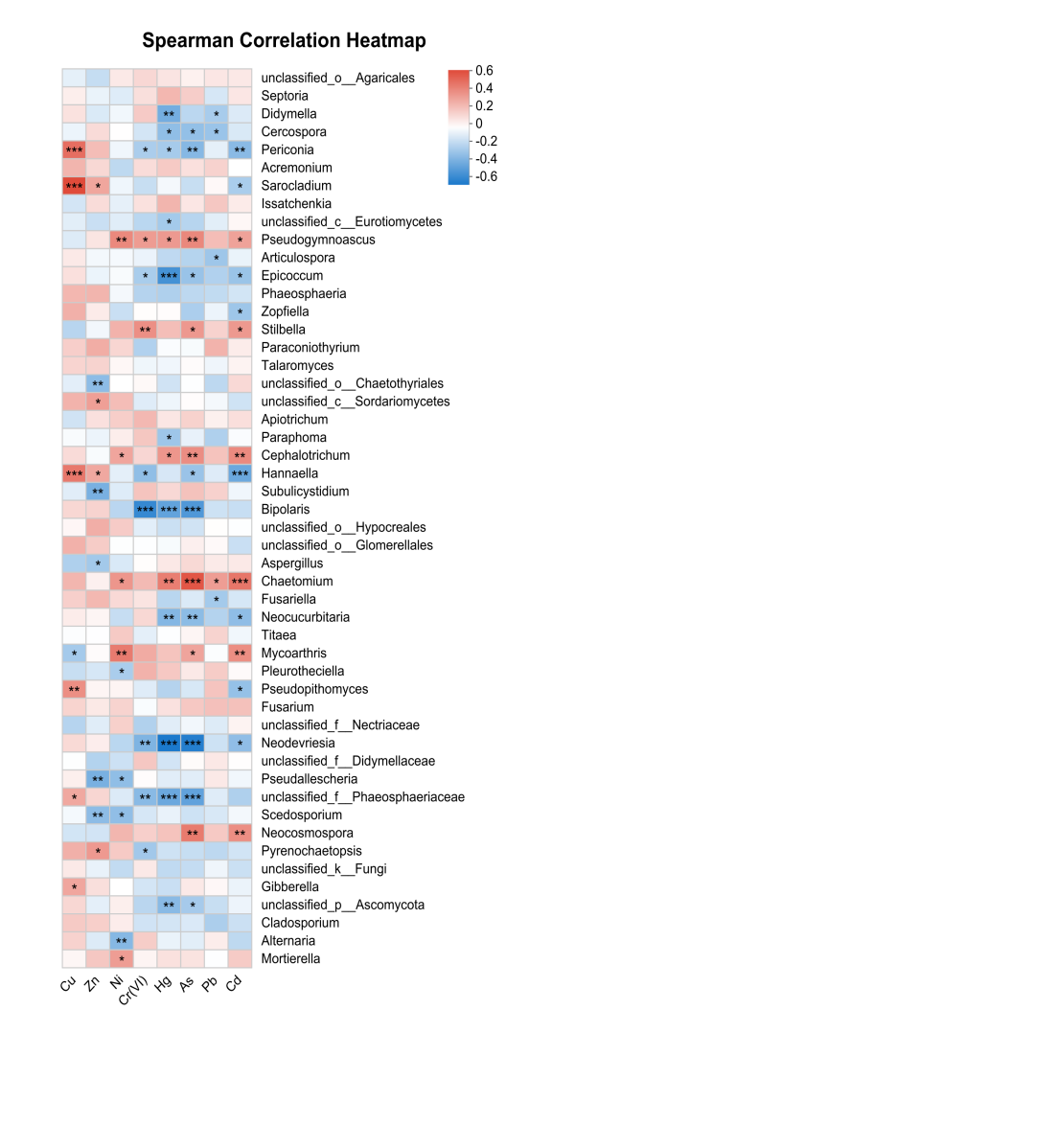


(a)

(b)

**Figure S10** The Spearman correlation analysis between soil microbial community and soil heavy metals. (a) was related to bacterial community, (b) was related to fungal community


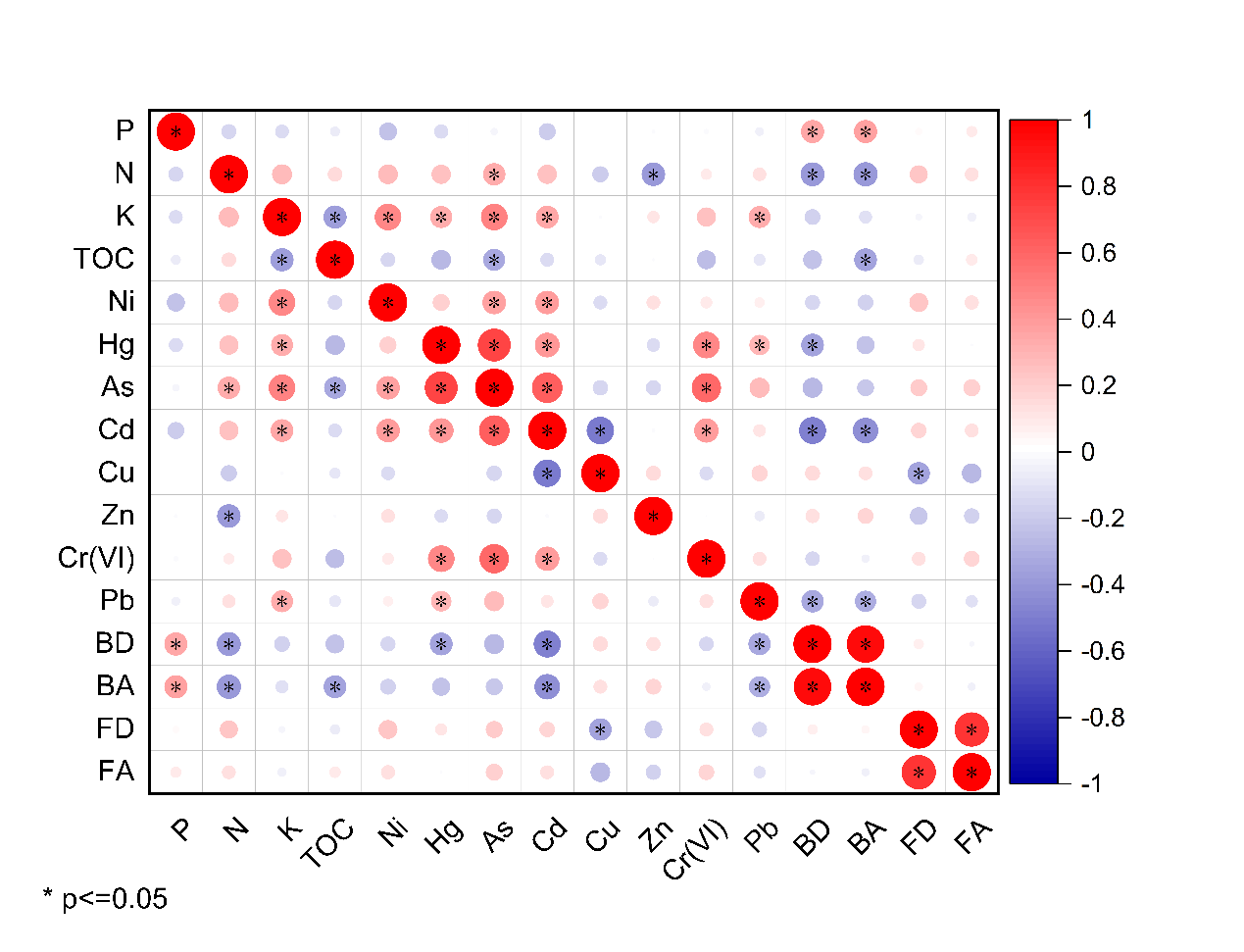


**Figure S11** The Pearson correlation analysis between potential explanatory variables (VIF<10) and microbial diversity (BD: bacterial diversity index, BA: bacterial richness index, FD: fungal diversity index, FA: fungal richness index).

**
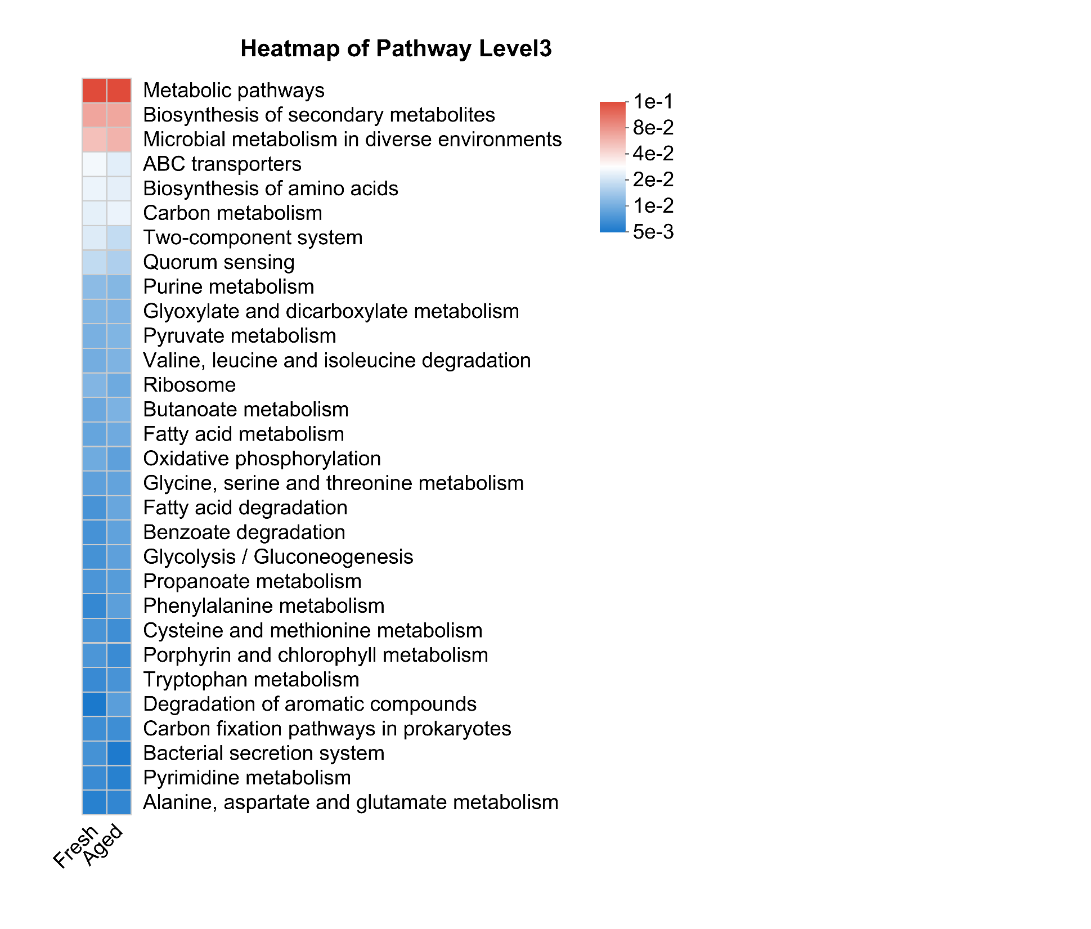
**

**Figure S12** The bacterial metabolic pathways in soil samples around aged and fresh landfill area predicted by PICRUSt2.

**
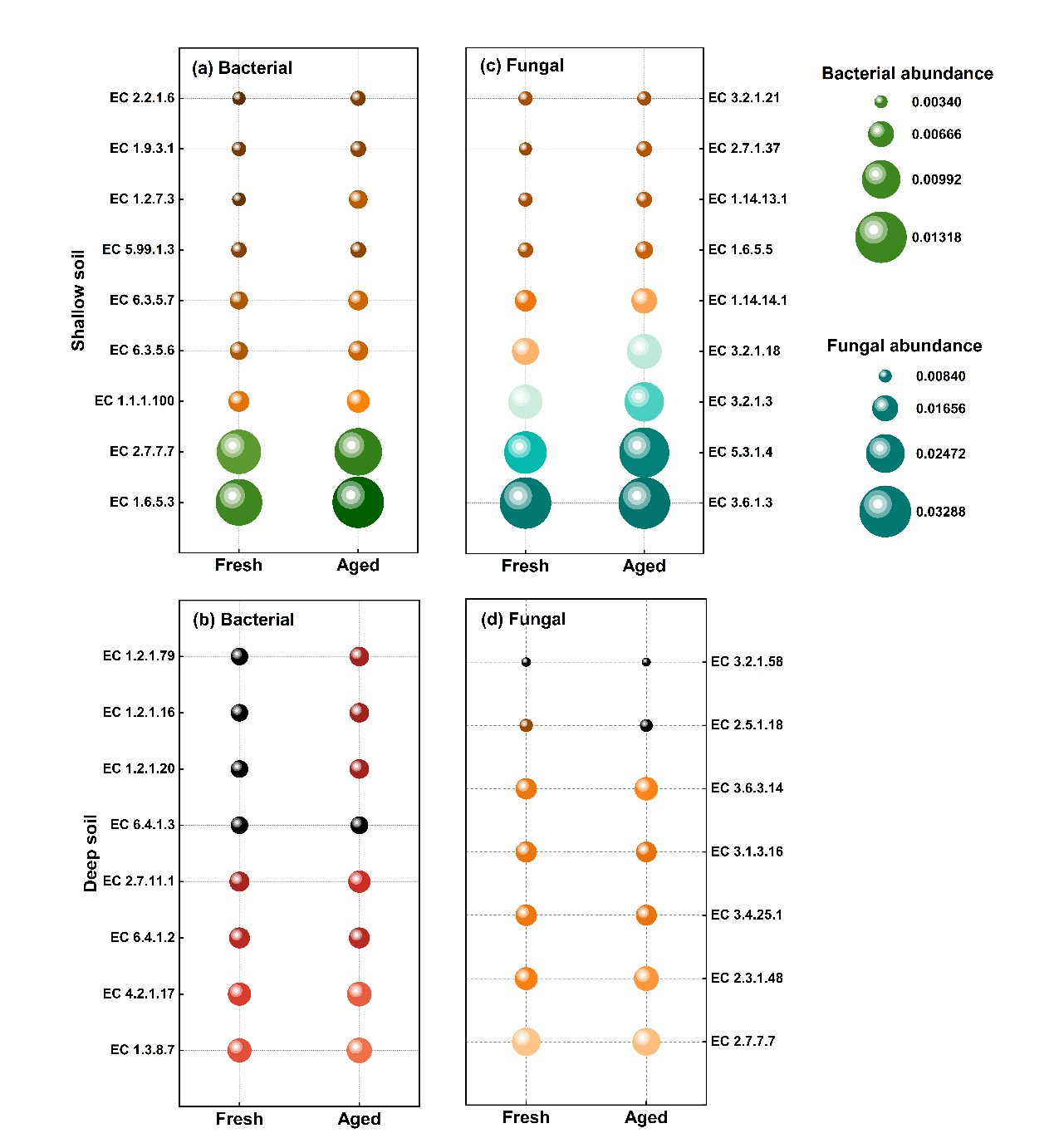
**

**Figure S13** Functional genes prediction of microbial contributor to changes in soil nutritional content. (a) bacterial genes in shallow soil around fresh and aged dumps, (b) bacterial genes in deep soil around fresh and aged dumps, (c) fungal genes in shallow soil around fresh and aged dumps, (d) fungal genes in deep soil around fresh and aged dumps.
